# *Drosophila* Spidey/Kar Regulates Oenocyte Growth via PI3-Kinase Signaling

**DOI:** 10.1371/journal.pgen.1006154

**Published:** 2016-08-08

**Authors:** Einat Cinnamon, Rami Makki, Annick Sawala, Leah P. Wickenberg, Gary J. Blomquist, Claus Tittiger, Ze'ev Paroush, Alex P. Gould

**Affiliations:** 1 The Francis Crick Institute, Mill Hill Laboratory, Mill Hill, London, United Kingdom; 2 Department of Biochemistry and Molecular Biology, University of Nevada, Reno, Reno, Nevada, United States of America; 3 Department of Developmental Biology and Cancer Research, Institute for Medical Research Israel Canada (IMRIC), Faculty of Medicine, the Hebrew University, Jerusalem, Israel; University of California San Francisco, UNITED STATES

## Abstract

Cell growth and proliferation depend upon many different aspects of lipid metabolism. One key signaling pathway that is utilized in many different anabolic contexts involves Phosphatidylinositide 3-kinase (PI3K) and its membrane lipid products, the Phosphatidylinositol (3,4,5)-trisphosphates. It remains unclear, however, which other branches of lipid metabolism interact with the PI3K signaling pathway. Here, we focus on specialized fat metabolizing cells in *Drosophila* called larval oenocytes. In the presence of dietary nutrients, oenocytes undergo PI3K-dependent cell growth and contain very few lipid droplets. In contrast, during starvation, oenocytes decrease PI3K signaling, shut down cell growth and accumulate abundant lipid droplets. We now show that PI3K in larval oenocytes, but not in fat body cells, functions to suppress lipid droplet accumulation. Several enzymes of fatty acid, triglyceride and hydrocarbon metabolism are required in oenocytes primarily for lipid droplet induction rather than for cell growth. In contrast, a very long chain fatty-acyl-CoA reductase (FarO) and a putative lipid dehydrogenase/reductase (Spidey, also known as Kar) not only promote lipid droplet induction but also inhibit oenocyte growth. In the case of Spidey/Kar, we show that the growth suppression mechanism involves inhibition of the PI3K signaling pathway upstream of Akt activity. Together, the findings in this study show how Spidey/Kar and FarO regulate the balance between the cell growth and lipid storage of larval oenocytes.

## Author Summary

Lipids play diverse roles in health and disease. Some types of lipids function as metabolic fuels for energy homeostasis, whereas others act as components of cell membranes or serve as signals regulating cell behaviors. Much, however, remains to be discovered about the molecular connections between different categories of lipids. Phosphatidylinositide 3-kinase (PI3K) is an enzyme that synthesizes phosphatidylinositide lipids, which act as signals essential for growth during normal development and cancer. Using genetics in the fruit fly, *Drosophila*, we identify new regulatory links between phosphatidylinositides and lipid oxidoreductases in specialized fat-metabolizing cells called oenocytes. We find that an enzyme metabolizing very long chain fatty acids (VLCFAs) and also a putative lipid dehydrogenase/reductase both act to prevent the inappropriate overgrowth of oenocytes. In the case of the latter enzyme, it suppresses cell growth by inhibiting phosphatidylinositide signaling. Future studies will determine whether similar lipid enzymes regulate PI3K signaling in other cell and tissue types during normal development and tumorigenesis.

## Introduction

The regulation of cell growth is fundamentally important for a wide range of biological processes [reviewed in 1, 2, 3]. A key signal transduction network regulating cell growth and proliferation in response to nutrients involves two related kinases, Target-of-Rapamycin (TOR) and Class I phosphatidylinositol 3-kinase (PI3K) [4–7]. A variety of nutritional and growth factor stimuli are known to activate PI3K, which converts the membrane phospholipid phosphatidylinositol-4,5-bisphosphate (PIP2) into phosphatidylinositol-3,4,5-trisphosphate (PIP3) [8]. Levels of PIP3 are kept in check by Phosphatase and Tensin Homologue (PTEN), which hydrolyzes PIP3 back to PIP2. PIP3 is a signaling lipid that stimulates plasma membrane recruitment of proteins with PIP3-specific pleckstrin homology (PH) domains such as Akt (also known as protein kinase B) and phosphoinositide-dependent kinase 1 (PDK1). The colocalization of Akt and PDK1 at the membrane surface increases the rate at which PDK1 phosphorylates Akt at a regulatory site essential for its activation [9]. Activated Akt is then able to phosphorylate numerous targets in the TOR/PI3K network, including Forkhead box subgroup O (FoxO) transcription factors and Tuberous Sclerosis Complex 2 (TSC2), an inhibitor of TOR [reviewed in 10]. Akt phosphorylation of both of these negative growth regulators attenuates their activities, thus promoting an increase in cell growth and biomass.

*Drosophila melanogaster* provide a useful genetic system for studying cell growth and PI3K signaling in the context of an intact organism. Tissue growth in *Drosophila*, as in mammals, depends upon the Insulin-like receptor (InR)/PI3K pathway and the interconnected amino-acid/TOR pathway [reviewed in 11, 12–15]. Class I PI3K is required for the growth of most if not all *Drosophila* tissues but the ways in which it is differentially regulated as a function of cell type and developmental stage are not yet fully clear. Nevertheless, some insights have been gained by experiments showing that there is selective tissue growth in larvae subjected to nutrient restriction (NR). At early larval stages, NR shuts down the growth of developing tissues, and prevents the stem cells of the central nervous system (neuroblasts) from re-entering the cell cycle after a period of quiescence [16–18]. At late larval stages, however, growth in neuroblast lineages is almost completely spared during NR, whereas it is approximately halved for the epithelial progenitors of adult structures (imaginal discs) and reduced to near zero in many other larval tissues [19, 20].

Among the larval tissues that are not spared during NR are two major organs of the *Drosophila* adipose axis: fat body cells (adipocytes) and oenocytes. The fat body provides the major storage depot in *Drosophila* for neutral lipids such as triglycerides, in the form of intracellular lipid droplets [21]. This tissue is important for the maintenance of energy homeostasis during starvation and acts as a nutrient sensor. Depending upon amino acid levels, the fat body can either store or release lipid nutrients into the hemolymph, in the form of lipoproteins [22–24]. Oenocytes are endocrine cells specialized for lipid metabolism [reviewed in 25, 26]. There are two morphologically distinct populations of *Drosophila* oenocytes, larval and adult (imaginal), each deriving from a separate pool of ectodermal progenitors [27–29]. Adult oenocytes synthesize species and sex-specific mixes of cuticular hydrocarbons that function in desiccation resistance and pheromonal communication [30–32]. These cuticular hydrocarbons are synthesized from very-long chain (VLC) fatty acids via a pathway requiring the cytochrome P450 enzyme Cyp4g1, a VLC fatty aldehyde decarbonylase [33, 34]. Larval oenocytes, on the other hand, are known to be essential for molting and synthesize VLC fatty acids required for waterproofing the tracheal system [35, 36]. Unlike most other cell types, larval oenocytes accumulate numerous lipid droplets during NR [35]. This oenocyte NR response resembles, at least superficially, the fasting-induced build up of neutral lipids (steatosis) observed in mammalian hepatocytes. In mammals, this steatosis is thought to be a physiological response to elevated lipolysis in adipose tissue. Similarly, in *Drosophila*, fat-body specific overexpression of an ortholog of Adipose Triglyceride Lipase (ATGL) is sufficient to induce steatosis in the oenocytes of fed larvae [35]. Hence, during NR, neutral lipid in the form of lipid droplets is lost from the fat body but gained by the oenocytes. The induction of lipid droplets during starvation requires the activity of the Lipophorin receptor (Lpr2) in oenocytes and so presumably involves the uptake of lipids released into the hemolymph [36]. It nevertheless remains unclear which tissue-specific signaling mechanisms allow neutral lipid content in the fat body and in oenocytes to be simultaneously regulated in opposite directions during starvation.

Here, we characterize novel regulatory interactions between PI3K signaling, lipid metabolism and cell growth in the context of the *Drosophila* adipose axis. Tissue-specific and clonal genetic analyses reveal that oenocytes respond to nutrition and PI3K signaling very differently from the fat body. PI3K signaling stimulates neutral lipid storage in fat body cells but it inhibits this process in oenocytes. We identify two lipid oxidoreductases that regulate the balance between lipid storage and cell size in oenocytes and show that one of these is part of an oenocyte-specific regulatory circuit that modulates PI3K-dependent cell growth.

## Results

### PI3K signaling inhibits lipid droplets in oenocytes but promotes them in fat body

**Fig 1:**
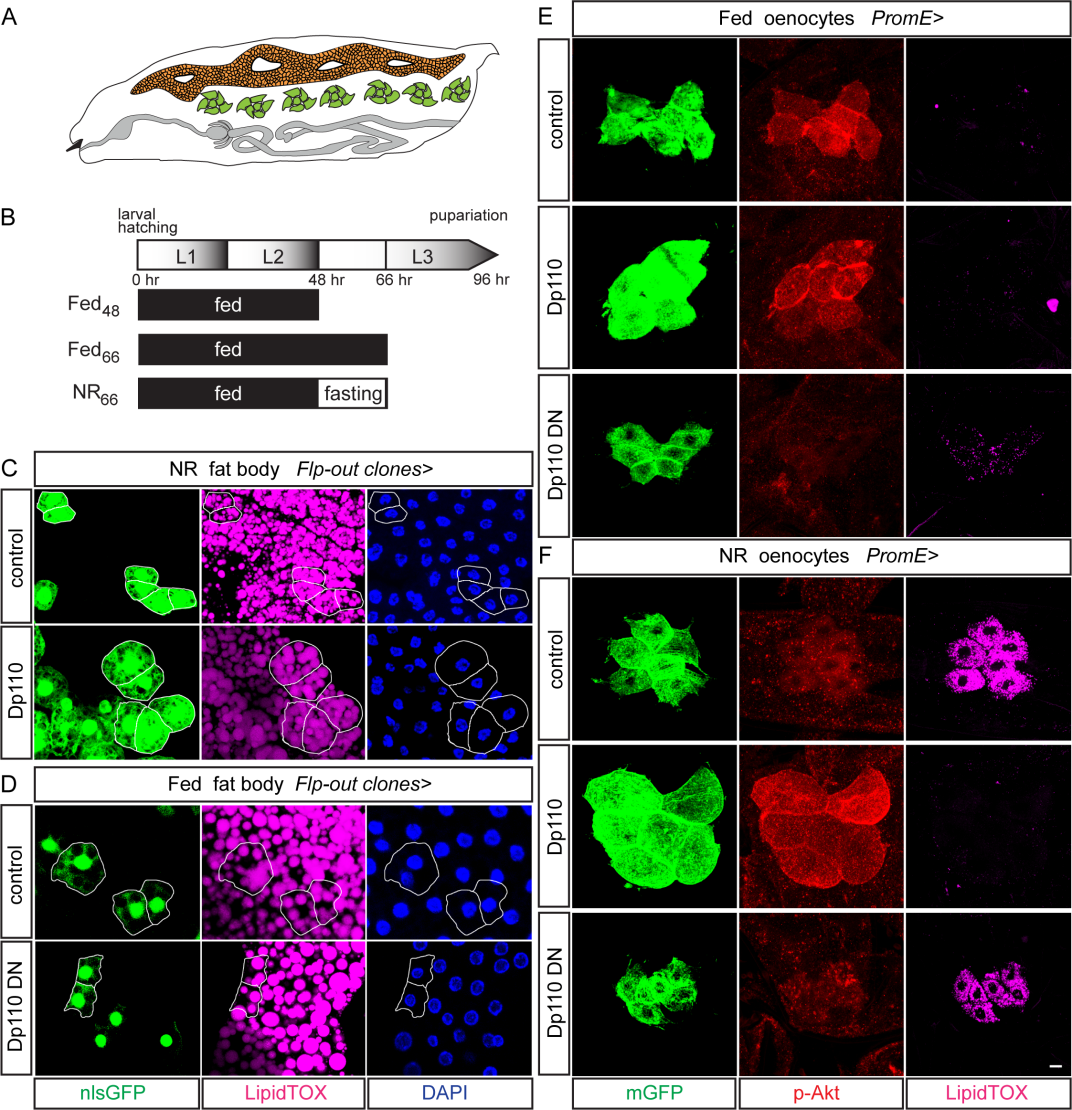
PI3K signaling inhibits lipid droplets in oenocytes but promotes them in fat body. (A) Cartoon of a *Drosophila* larva showing the principal organs of lipid metabolism: fat body (orange), oenocytes (green) and gut (gray). (B) Time line (0 hr to 96 hr) of development through the three larval instars (L1 to L3) with the experimental regimes of fed and nutrient restriction (NR) windows indicated beneath (Fed_48_, Fed_66_ and NR_66_). (C,D) Fat body flip-out clones marked with nlsGFP, nuclei stained with DAPI. Overexpressing PI3K (Dp110) in NR_66_ larvae (C) gives larger cells with larger lipid droplets (LipidTOX) than those in control clones. Expressing dominant-negative PI3K (Dp110^DN^) in Fed_48_ larvae (D) gives smaller cells with a similar density of lipid droplets (LipidTOX) as in control clones. (E, F) Oenocyte-cluster specific (*PromE-GAL4*) expression of wild-type (Dp110) or dominant-negative (Dp110^DN^) showing oenocyte morphology (mGFP), phospho-Akt staining (p-Akt) and lipid droplets (LipidTOX). In Fed_48_ larvae (E), oenocyte lipid droplets are largely absent but oenocyte size and membrane p-Akt expression are increased by Dp110 and decreased by Dp110^DN^ expression. In NR_66_ larvae (F), lipid droplets are abundant in control and Dp110^DN^ but not in Dp110 oenocyte clusters. NR oenocyte size is increased by Dp110 and decreased by Dp110^DN^. NR membrane p-Akt expression is dramatically increased in Dp110, compared to control larvae and Dp110^DN^-expressing oenocytes. Scale bar is 10 μm. In this and all subsequent figures, confocal images are projections of several sections unless stated otherwise. See S1 and S2 Figs for quantitations.

When raised on an optimal diet, *Drosophila* larvae grow (increase mass) by more than two orders of magnitude as they develop through three instars (L1-L3) over a four-day (~96 hr) period. Fat body and oenocytes (Fig 1A), in common with many other larval tissues, grow via an increase in cell size and ploidy rather than by cell division [37, 38]. We first compared how oenocytes and fat body respond to PI3K signaling. To assess the cell-autonomous effects of class I PI3K signaling upon fat body cell size and intracellular lipid droplets, genetic mosaic larvae were generated via Flp/FRT mediated activation of the GAL4/UAS system (Flp-out clones). A previous study found that overexpressing PI3K (UAS-Dp110) in Flp-out clones in the fat body is sufficient to increase cell size in well-fed larvae [39]. During starvation it is known that neutral lipids, stored in intracellular lipid droplets, decrease in the fat body yet increase in oenocytes and neither tissue is able to grow significantly [19, 35]. To investigate the role of PI3K signaling in the regulation of lipid droplets during nutrient restriction (NR), larvae at the early-L3 stage (48hr after larval hatching) were switched from fed (yeast/cornmeal/agar) to NR (PBS/agarose) medium for 18 hr. Tissues were analyzed either at the early L3 stage (Fed_48_ control group) or 18 hr later (NR_66_ experimental and Fed_66_ control groups) (Fig 1B). NR applied at early L3 induces developmental arrest such that, for NR_66_ larvae, their chronologically matched control is Fed_66_ but their developmentally matched control is Fed_48_. Figures therefore show comparisons between Fed_48_ and NR_66_ larvae, although in most cases the Fed_66_ time point was also analyzed. Following 18 hr of NR, the size of fat body cells is decreased but they are only partially depleted of stored neutral fat and so still contain numerous lipid droplets [23, 35 and Fig 1C]. Overexpression of PI3K resulted in increases in fat body cell size, lipid droplet diameter and neutral lipid content following NR (Figs 1C, S1A and S2A). The converse manipulation, expressing a dominant-negative PI3K (Dp110^DN^) resulted in the expected decrease in fat body cell size but it did not significantly alter lipid droplet content in fed conditions (Figs 1D, S1B and S2B). These results together suggest that a decrease in PI3K signaling is needed for neutral lipid loss from the fat body during NR but that this change alone is not enough to drive lipid loss in the fed state.

We next manipulated PI3K activity in oenocytes using a larval oenocyte-specific GAL4 driver (*PromE-GAL4*, [31, 36]). To monitor PI3K signaling levels during these manipulations, we used an antibody recognizing phospho-Akt (Ser505). Although technically challenging to detect, membrane localization of phospho-Akt provides a readout for most Akt activity [19, 40, 41]. Control GFP-labeled oenocyte clusters showed higher and more consistent expression of membrane phospho-Akt in Fed_48_ than in NR_66_ larvae, consistent with the known nutrient-dependence of PI3K/TOR signaling (Fig 1E and 1F). GFP-labeled oenocyte clusters with overactive PI3K signaling (*PromE>Dp110*) are very large in size in both Fed_48_ and NR_66_ larvae and this "giant oenocyte" phenotype is associated with increased expression and membrane localization of phospho-Akt (Figs 1E, 1F and S2C–S2F). Increased oenocyte size was also observed using a second method for boosting PI3K activity: expressing an activated myristoylated form of Akt (myr-Akt) in Flp-out clones (S2G Fig). Conversely, we found that dominant-negative PI3K (*PromE>Dp110*^*DN*^) decreases oenocyte size in both Fed_48_ and NR_66_ larvae and, in the latter context, p-Akt staining remains detectable, although weak and close to the limit of detection (Figs 1E, 1F and S2C–S2F). A similar cell size decrease is also observed in Flp-out clones expressing *Dp110*^*DN*^ (S2G Fig). During NR, we observed that oenocytes not only express weak p-Akt but they also retain higher expression of a GFP sensor for PIP3 levels [tGPH, 39] and weaker nuclear FoxO expression than do fat body cells (S3 Fig). Oenocytes therefore sustain low-level PI3K signaling during NR. Nevertheless, decreasing this low level even further using *Dp110*^*DN*^ does not significantly block the normal 100-fold increase in lipid droplets during NR, suggesting that it is not an obligate positive input (Figs 1F and S1C–S1E). Rather, PI3K signaling in oenocytes has primarily a negative input as *Dp110*^*DN*^ induces a low level of lipid droplets in the fed state and *Dp110* overexpression efficiently blocks them during NR (Figs 1E, 1F, S1D and S1E). In the context of adult oenocytes, it was reported that lipid droplet induction during starvation requires Insulin-like receptor activation via Ilp6 secreted from the adult fat body [42]. For larval oenocytes, however, we find that Ilp6 is not required for lipid droplet induction during NR (S4A and S4B Fig). Together, the results thus far demonstrate that artificial overactivation of PI3K is sufficient to stimulate NR growth of both cell types in the larval adipose axis but that it has opposite effects on NR fat storage in lipid droplets: inhibiting it in oenocytes yet promoting it in the fat body.

### Many lipid metabolic enzymes regulate lipid droplets but not oenocyte size

**Fig 2:**
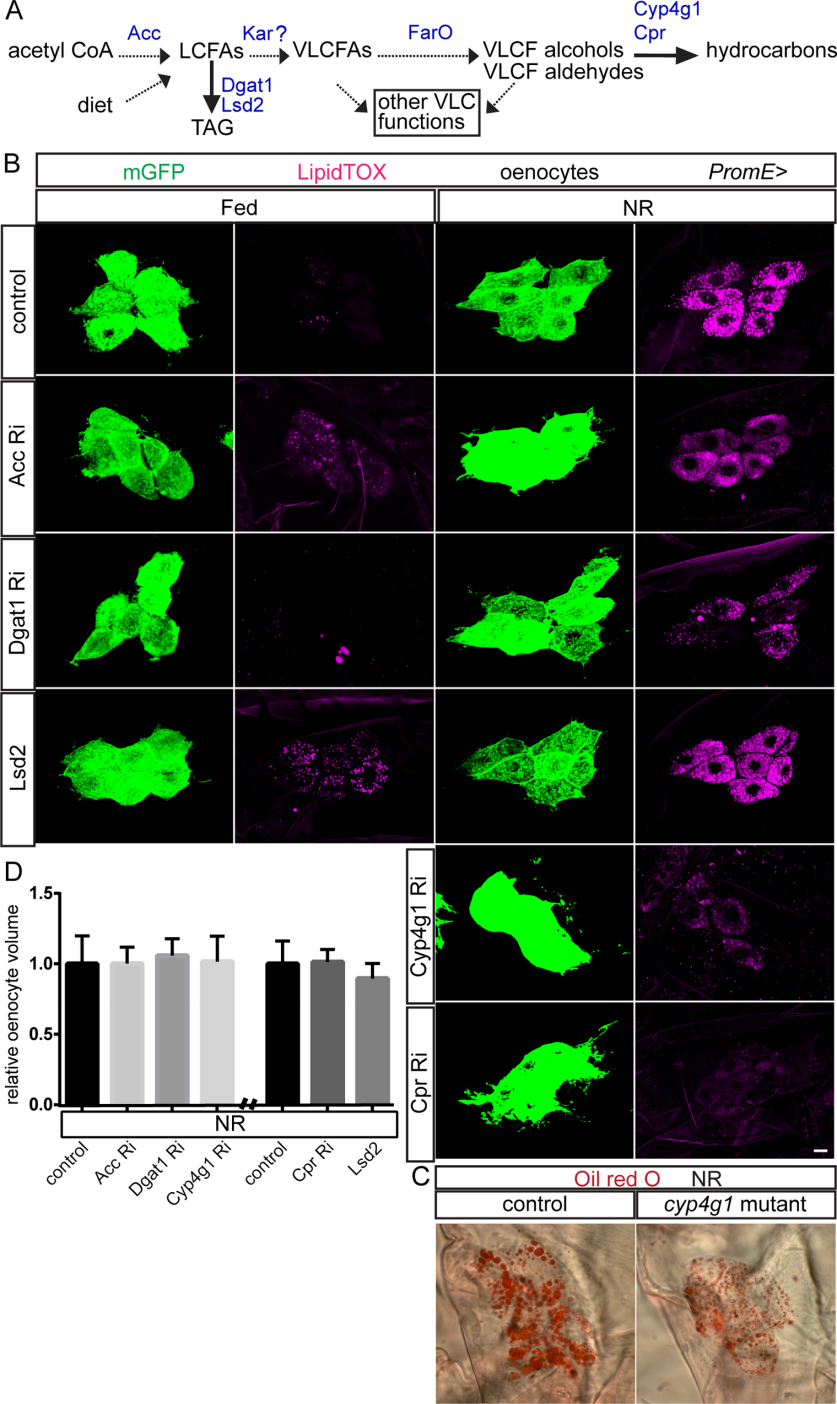
Fatty acid, triglyceride and hydrocarbon metabolism regulate oenocyte lipid droplets. (A) Simplified presentation of the pathway for long chain (LCFA) and very long chain (VLCFA) fatty acid, triglyceride (TAG) and hydrocarbon synthesis. See text for details of the *Drosophila* enzymes (blue) analyzed in this study. The questionmark indicates that the enzyme activity of Kar has not yet been directly established. (B) Oenocyte clusters (mGFP labeled) from Fed_48_ and NR_66_ larvae expressing *PromE-GAL4* driven *UAS-RNAi* for *Acc*, *Dgat1*, *Cyp4g1* or *Cpr* indicating decreased or blocked induction of lipid droplets (LipidTOX) during NR. *PromE-GAL4* driven *UAS-Acc RNAi* or *UAS-Lsd2* are the only manipulations that gave a modest increase in oenocyte lipid droplets in Fed_48_ larvae. Scale bar is 10 μm (C) Oenocyte cluster from a *Cyp4g1*^*Δ4*^ hemizygous larva (*Cyp4g1* mutant) showing a decrease in lipid droplet (Oil Red O) induction during NR. (D) Graph of relative oenocyte volumes for the five genetic manipulations in B showing no significant changes in cell size in NR_66_ larvae. In this and subsequent graphs, error bars represent 1 s.d. and asterisks show statistical significance in Student t tests (*p<0.05, and **p<0.001) compared to the black control bar unless otherwise indicated. See S1 Fig for quantifications.

Before determining how PI3K signaling inhibits lipid droplets in a tissue-specific manner, we first investigated the oenocyte droplet induction mechanism itself. We focused on the metabolism of VLCFAs as these are known to be synthesized selectively in larval oenocytes [35, 36 and Fig 2A]. Consistent with a previous study [36], in Fed_48_ larvae, *PromE-GAL4* driven RNAi knockdown of the malonyl-CoA synthesizing enzyme Acetyl-CoA Carboxylase (Acc) significantly increased oenocyte lipid droplets (Figs 2B and S1F). This fed increase reflects an amount of lipid droplets per oenocyte that is considerably lower than that observed during NR in control larvae. It is known to be mediated via the uptake of fatty acids in fed larvae via Lpr2, a Lipophorin receptor [36]. Unexpectedly, in NR larvae, we observed that Acc knockdown decreased lipid droplet induction, suggesting that fatty acid synthesis makes a contribution to starvation-induced oenocyte steatosis (Figs 2B and S1G). In a previous study, *Acc* knockdown in oenocytes was also associated with tracheal flooding and systemic hypoxia [36]. Importantly, however, using our genetic and dietary conditions, the majority of larvae expressing *PromE-GAL4* driven *UAS-RNAi* of *Acc* or other genes in this study do not display tracheal flooding even though knockdown efficiencies are over 90% (S4C and S5 Figs). As the PromE knockdown phenotypes described in our study are observed in most if not all larvae, they are highly unlikely to be a secondary consequence of hypoxia due to flooding of the trachea. Similar to Acc, knockdown of Diacylglycerol acyltransferase 1 (DGAT1), a dedicated enzyme in the synthesis of TAGs [43], gave a decrease in lipid droplet induction during NR in oenocytes (Figs 2B, S1F and S1G). Conversely, *PromE* overexpression of Lsd-2 (perilipin-2), a protein that inhibits lipolysis to promote TAG storage in lipid droplets [44], led to a statistically significant increase in oenocyte lipid droplets in Fed_48_ but not in NR larvae (Figs 2B, S1F and S1G). This suggests that there is active lipolysis in the fed state and that this may help to prevent the accumulation of lipid droplets. Cytochrome P450 4g1 (Cyp4g1), with its redox partner Cytochrome P450 reductase (Cpr), together form a microsomal holoenzyme involved in converting VLCFAs into hydrocarbons in the oenocytes of adults [33]. Strikingly, RNAi knockdowns for either enzyme or a *Cyp4g1* loss-of-function mutation [35] attenuated lipid droplet induction during NR but had little effect in Fed_48_ larvae (Figs 2B, 2C and S1F–S1H). These results strongly suggest that the Cyp4g1/Cpr holoenzyme is required for the induction of lipid droplets in larval oenocytes during NR. We next tested whether any of the above lipid biosynthetic enzymes regulating oenocyte lipid droplets might also control oenocyte size during NR. However, no significant differences in oenocyte volume were observed with *PromE* driven overexpression of Lsd2 or RNAi knockdown of Acc, Dgat1, Cyp4g1 or Cpr (Fig 2D). Together with the previous results, this demonstrates regulation of oenocyte lipid droplets, not only by PI3K signaling but also by triglyceride and hydrocarbon biosynthetic enzymes. In contrast, regulation of oenocyte cell size is sensitive to PI3K signaling but not to the five tested triglyceride and hydrocarbon biosynthetic enzymes.

### Oenocyte Kar and FarO promote lipid droplets and suppress cell growth

**Fig 3:**
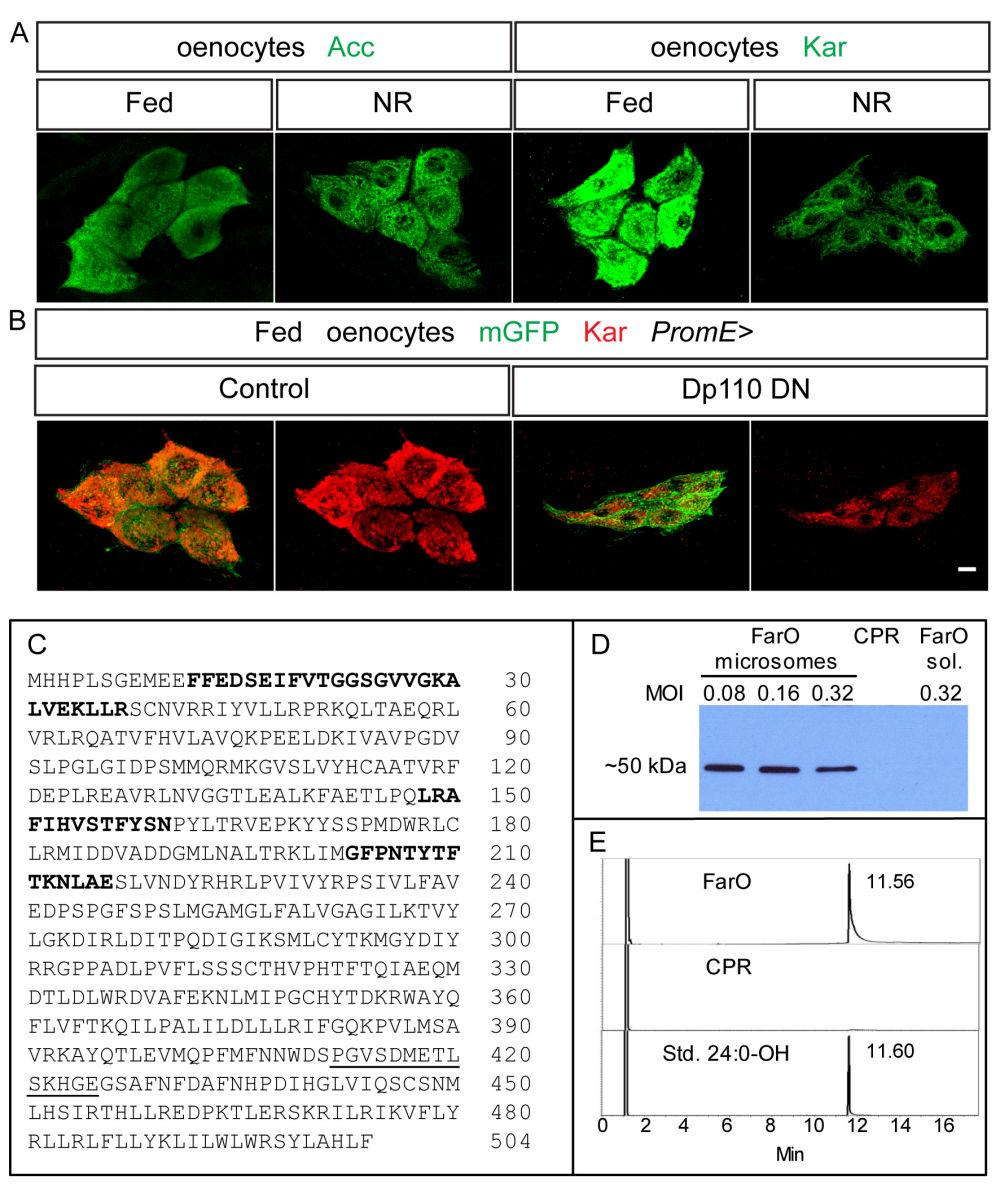
Kar is regulated by nutrients and FarO is a VLC fatty acyl-CoA reductase. (A,B) Nutrients and PI3K signaling are required for maximal Kar expression. (A) Kar protein is expressed more strongly in oenocytes from Fed_48_ than from NR_66_ larvae, whereas Acc protein levels do not change so noticeably. (B) Kar protein levels decrease in Fed_48_ larvae following inhibition of PI3K activity (*PromE>Dp110*^*DN*^). (C) FarO amino acid sequence showing three regions (bold) incorporating the diagnostic Rossman Fold and YXXXK containing catalytic motif and the peptide used to produce polyclonal antisera (underlined). (D) Western blot of microsomes or supernatants (sol.) of Sf9 cell lysates infected with recombinant baculovirus encoding either FarO or housefly CPR at different multiplicities of infection (MOI), probed with anti-FarO antibody. (E) GC traces of extracts from functional assays of recombinant FarO or CPR (negative control) incubated with 24:0-CoA. Reactions with FarO yielded a product with an identical migration to that of a 24:0 alcohol standard.

We next extended the oenocyte genetic analysis to two enzymes, Kar and FarO, which are not dedicated to triglyceride or hydrocarbon production but to more general roles in the synthesis of VLCFAs, VLCF aldehydes and VLCF alcohols (Fig 2A). *CG1444* encodes a protein with YXXXK catalytic and putative NADH binding motifs found in many enzymes of the short-chain reductase/dehydrogenase (SDR) family [45]. It has been reported to encode the only predicted 3-ketoacyl-CoA reductase (Kar) in the *Drosophila melanogaster* genome and is required in larval oenocytes for the synthesis of a putative VLCFA implicated in tracheal waterproofing and also in adult oenocytes for the synthesis of VLCFA-derived cuticular hydrocarbons [34, 36]. However, the activities and substrate specificities of the Kar enzyme (also known as Spidey) have yet to be directly determined. We raised an antibody against Kar and observed that the levels of Kar protein are substantially attenuated but nevertheless remain expressed in oenocytes during NR, whereas Acc levels do not noticeably change between fed and NR (Fig 3A). Kar protein levels are also moderately decreased when PI3K signaling is inhibited in the oenocytes of Fed_48_ larvae (Fig 3B). Hence, dietary nutrients and PI3K signaling not only regulate lipid droplet accumulation in oenocytes but also Kar expression. Another enzyme of VLCFA metabolism, fatty acyl-CoA reductase, converts fatty acyl-CoA esters into fatty alcohols. *CG18031* encodes one of several predicted fatty acyl-CoA reductases in *Drosophila*. It is selectively expressed in oenocytes [35] and will therefore be referred to as *FarO*. It encodes a protein with a Rossmann–fold NAD(P)H binding domain with a [ST]GXXGXXG motif and also a YXXXK active site motif as found in other fatty–acyl–CoA reductases (Fig 3C). *Drosophila* FarO produced from a recombinant baculovirus in Sf9 cells is a microsomal enzyme that, in the presence of NADH or NADPH, can convert tetracosanoic-CoA ester (C24:0-CoA) to a product with an elution time identical to that of tetracosanol (C24:0) (Fig 3D and 3E). Recombinant FarO also produced alcohols from C26:0-CoA, as well as from C26:0 and C28:0 fatty acids pre-incubated with CoA-SH. These biochemical experiments demonstrate that FarO is a fatty acyl-CoA reductase that reduces VLCFA-CoAs to VLCF alcohols.

**Fig 4:**
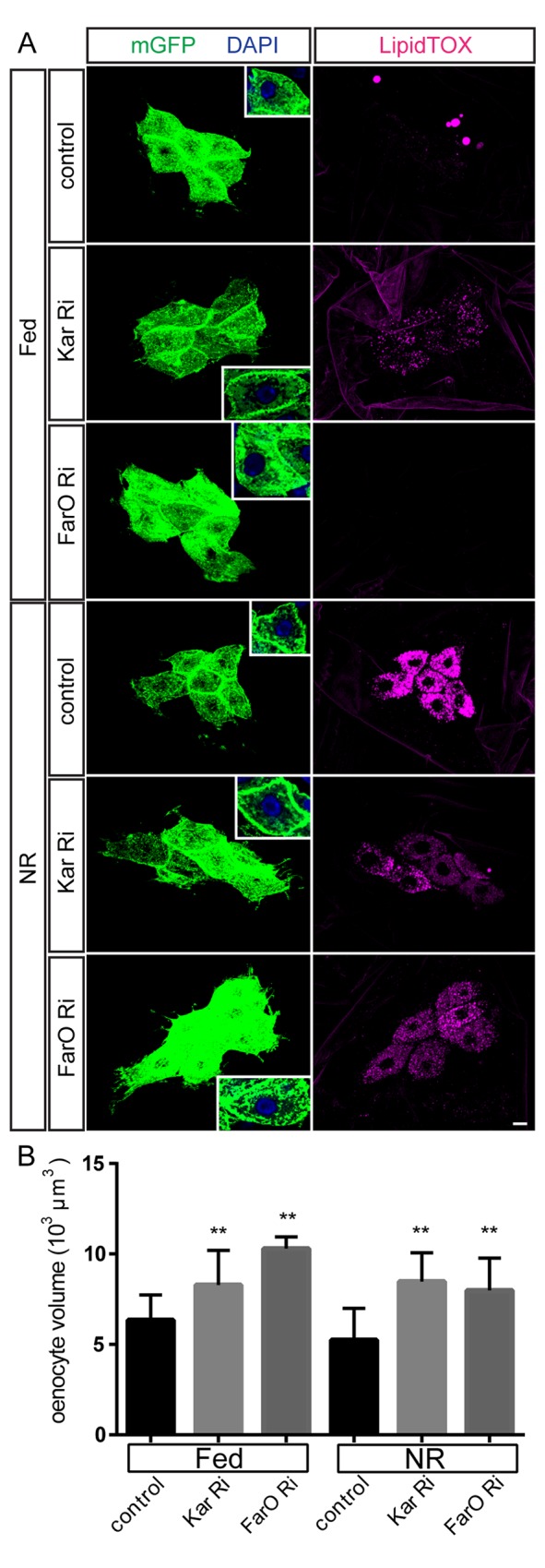
Kar and FarO decrease oenocyte size and stimulate lipid droplet induction. (A) Oenocyte-specific RNAi knockdown (*PromE-GAL4*) for Kar or FarO in oenocytes increases their cell size in both Fed_48_ and NR_66_ larvae (insets) and markedly decreases the induction of lipid droplets (LipidTOX) in NR larvae. Note that oenocytes from Fed_48_ Kar RNAi larvae also display a small increase in lipid droplets, as is also seen with Acc RNAi. Scale bar is 10μm (B) Graph showing quantitation of larval oenocyte volumes and a significant (**p<0.001) increase in Fed_48_ and NR cell volumes for Kar RNAi and FarO RNAi. See S1 Fig for quantitations.

We next tested the effects of knocking down Kar or FarO with RNAi. In the fat body, RNAi Flp-out clones for Kar or FarO did not detectably alter lipid droplets or cell size during NR (S6A Fig). In oenocytes, however, RNAi knockdown of either gene did give a phenotype using *PromE-GAL4* or a second tissue-selective driver (see Materials and Methods). Knockdown of Kar but not FarO was associated with a significant increase of lipid droplets in the fed state, to a low level similar to that observed with Acc knockdown (Figs 4A and S1I). Nevertheless, the knockdown of either Kar or FarO strongly decreased the accumulation of oenocyte lipid droplets during NR (Figs 4A and S1J). Using a second genetic method, Flp-out clones, FarO RNAi was also observed to abrogate oenocyte droplet induction (S6B and S6C Fig). Together, these findings demonstrate that the lipid reductases/dehydrogenases Kar and FarO are both required in oenocytes for the induction of lipid droplets during NR. Strikingly, we also observed that RNAi knockdown of either Kar or FarO with *PromE-GAL4* markedly increases the volume of Fed_48_ or NR_66_ oenocytes (Fig 4A insets and 4B). Control and knockdown oenocytes are similar in size prior to larval hatching (0 hr) so excessive oenocyte size likely reflects a greater volume gain (i.e growth rate) during the first 48 hr of larval development. However, by 66 hr (Fed_66_), Kar and FarO knockdown oenocyte sizes are no longer significantly different from controls, even though oenocyte size remains PI3K-dependent at this later developmental stage (S6D and S6E Fig). Given that controls catch up in size with knockdown oenocytes by 66hr, it may be that Kar or FarO RNAi do not alter final oenocyte size at the end of larval development (96 hr). We conclude more generally that, even though many oenocyte enzymes involved in triglyceride and hydrocarbon biosynthesis do not influence cell size, the two lipid oxido reductases Kar and FarO both play a key role in preventing excessive cell growth during early larval development.

### Kar regulates oenocyte nuclear size and endoreplication

**Fig 5:**
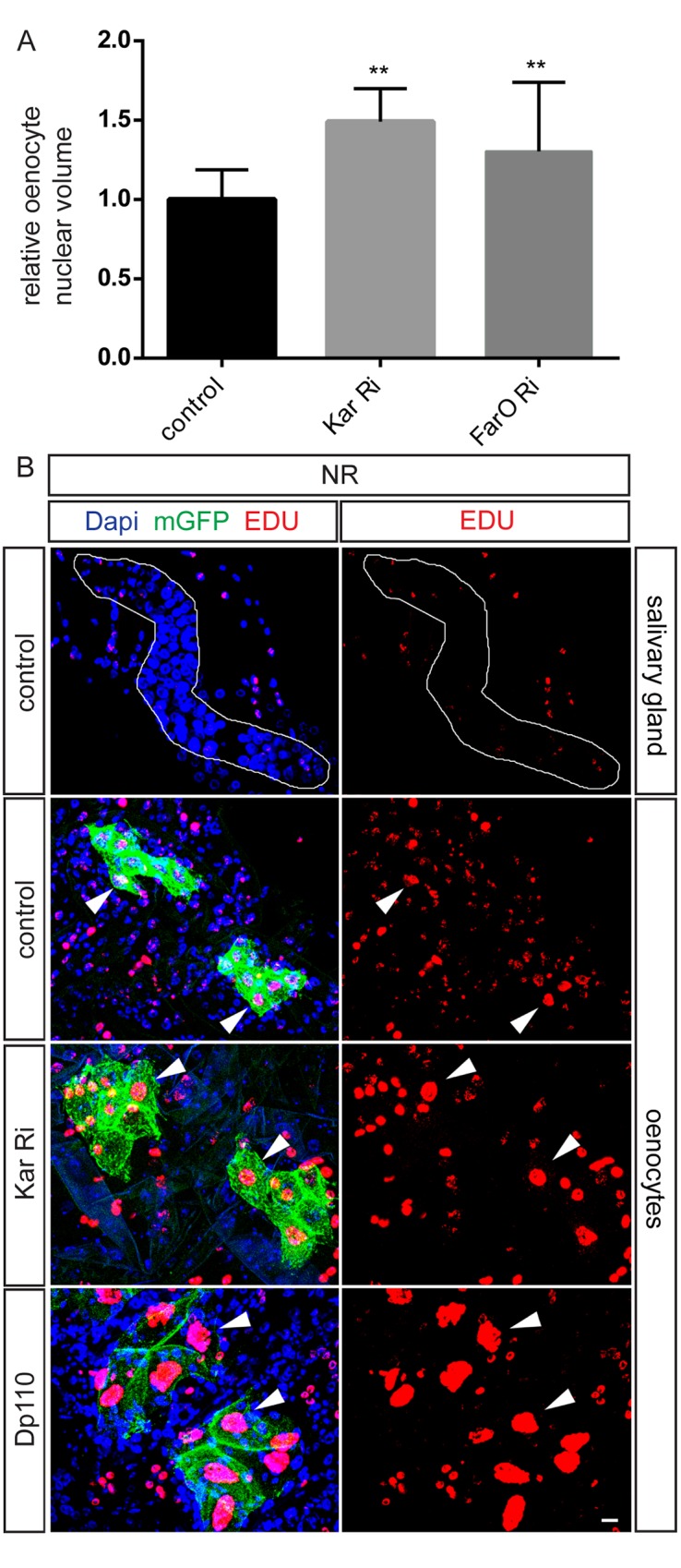
Kar is required to restrain oenocyte endoreplication. (A) *PromE-GAL4* driven Kar RNAi or FarO RNAi in NR larvae leads to a significant (**p<0.001) increase in oenocyte nuclear volume. (B) *PromE-GAL4* driven Kar RNAi or PI3K overexpression (Dp110) in NR larvae increases endoreplication (EdU nuclear incorporation) in oenocytes (marked with mGFP). Note that in larvae of the control genotype, endoreplication is shut down by NR in almost all cells of the salivary gland (outlined in white) but not in all oenocytes. Examples of EdU-positive oenocytes are indicated (white arrowheads). Scale bar is 10 μm.

Kar and FarO could potentially regulate oenocyte size via several distinct mechanisms, such as osmotic swelling/shrinkage or the cell cycle. The developmental growth of many larval cell types, including oenocytes, is known to be accompanied by rounds of DNA replication without cytokinesis that lead to polyploidy and increasing nuclear size [reviewed in 38]. Following RNAi knockdown of Kar or FarO, we observed that NR cell volume increases are concomitant with nuclear enlargement (Fig 5A). To visualize DNA replication directly, we then used the *in vivo* incorporation of the thymidine analogue 5-ethynyl-2'-deoxyuridine (EdU). Consistent with previous studies, EdU is incorporated into the polyploid nuclei of many mid-L2 tissues including oenocytes in fed larvae. In contrast, when L2 larvae were subjected to NR for 12 hr before exposure to EdU in NR medium, nuclear incorporation in many tissues such as the salivary gland is near zero whereas in the majority, although not all, oenocytes it is detectable (Fig 5B). This suggests that, unlike salivary gland cells, oenocytes retain a low-level of endoreplication for at least 12 hr during NR. Importantly, EdU incorporation in oenocyte nuclei during NR is markedly increased in the enlarged oenocytes generated by Kar knockdown (*PromE>Kar Ri*) and can be even further increased in the giant oenocytes resulting from strong PI3K hyperactivation (*PromE>Dp110*) (Fig 5B). Kar therefore regulates a *bona fide* growth mechanism that increases both cell size and nuclear ploidy. Moreover, the opposing effects of Kar and PI3K upon oenocyte size correlate with their effects upon the oenocyte endocycle.

### Kar inhibits p-AKT and PI3K signaling to limit oenocyte growth

**Fig 6:**
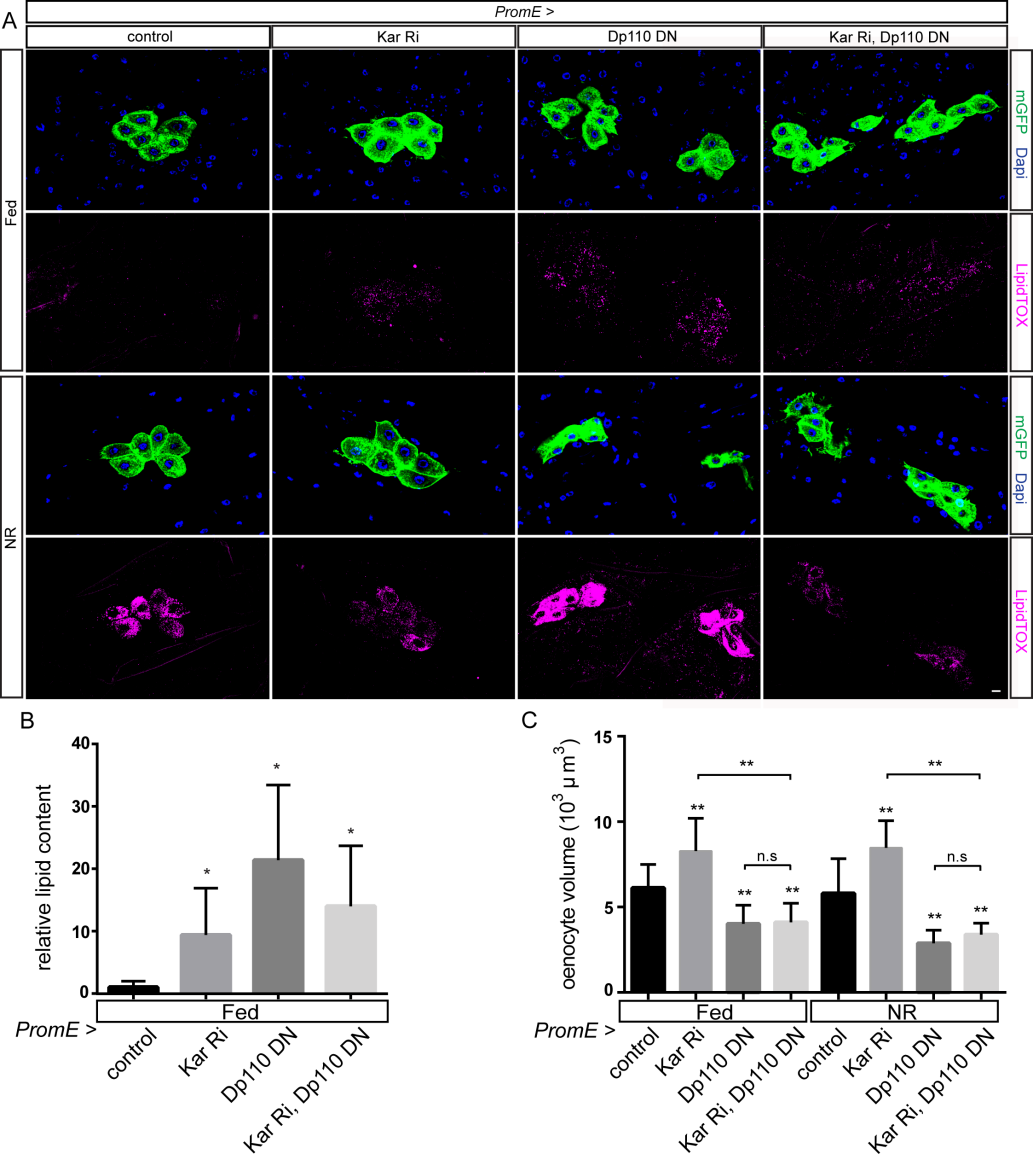
Genetic interactions between Kar and PI3K. (A) In Fed_48_ larvae, both *PromE>Kar Ri* and *PromE>Dp110*^*DN*^ genotypes accumulate low levels of oenocyte lipid droplets and this is also seen in *PromE>Kar Ri Dp110*^*DN*^. In NR larvae, *PromE>Dp110*^*DN*^ does not noticeably alter lipid droplet induction and thus inhibition of NR droplet induction is similar in *PromE>Kar Ri* and in *PromE>Kar Ri Dp110*^*DN*^. Oenocyte volume in both Fed_48_ and NR larvae is increased in *PromE>Kar Ri* but decreased in *PromE>Kar Ri Dp110*^*DN*^ to a similar extent as for *PromE>Dp110*^*DN*^ alone. Panels show single confocal sections and the scale bar is 10 μm. (B) Relative neutral lipid content of oenocytes in Fed_48_ larvae of the genotypes in panel A. (C) Oenocyte volumes of the genotypes in panel A in Fed_48_ and NR_66_ larvae, demonstrating that *PromE-GAL4* driving the expression of Dp110^DN^ is epistatic to Kar RNAi with respect to cell size. Statistical significance in Student t tests is indicated with asterisks (*p<0.05 and **p<0.001). See S1 Fig for LipidTOX quantifications.

To explore further the genetic interactions between lipid oxidoreductases and PI3K signaling, we made double combinations of Kar or FarO RNAi with dominant negative PI3K (*Dp110*^*DN*^). For oenocyte lipid droplets, double combinations were analyzed in the fed state as Dp110^DN^ does not have any significant effect in NR larvae. Kar knockdown and Dp110^DN^ each increase fed oenocyte lipid droplets to a moderate level and, not surprisingly, this was also observed when they were combined (Fig 6A and 6B). A different result was obtained with FarO knockdown, which efficiently blocked the Dp110^DN^ increase of lipid droplets seen in Fed_48_ larvae (S7A and S7B Fig). These results show that, in the context of oenocyte lipid metabolism, FarO and PI3K interact antagonistically. They also suggest that Kar and FarO interact differently with PI3K signaling. Genetic interactions of Kar and FarO with PI3K were also analyzed in the context of oenocyte size. For FarO knockdown, an intermediate oenocyte size phenotype was obtained with Dp110^DN^ precluding clear conclusions about regulatory connections (S7A and S7C Fig). For Kar knockdown, however, the small cell volume obtained with Dp110^DN^ was epistatic in both fed and NR larvae (Fig 6A and 6C). This shows that the increase in oenocyte size observed following Kar knockdown is strictly dependent upon PI3K activity.

**Fig 7:**
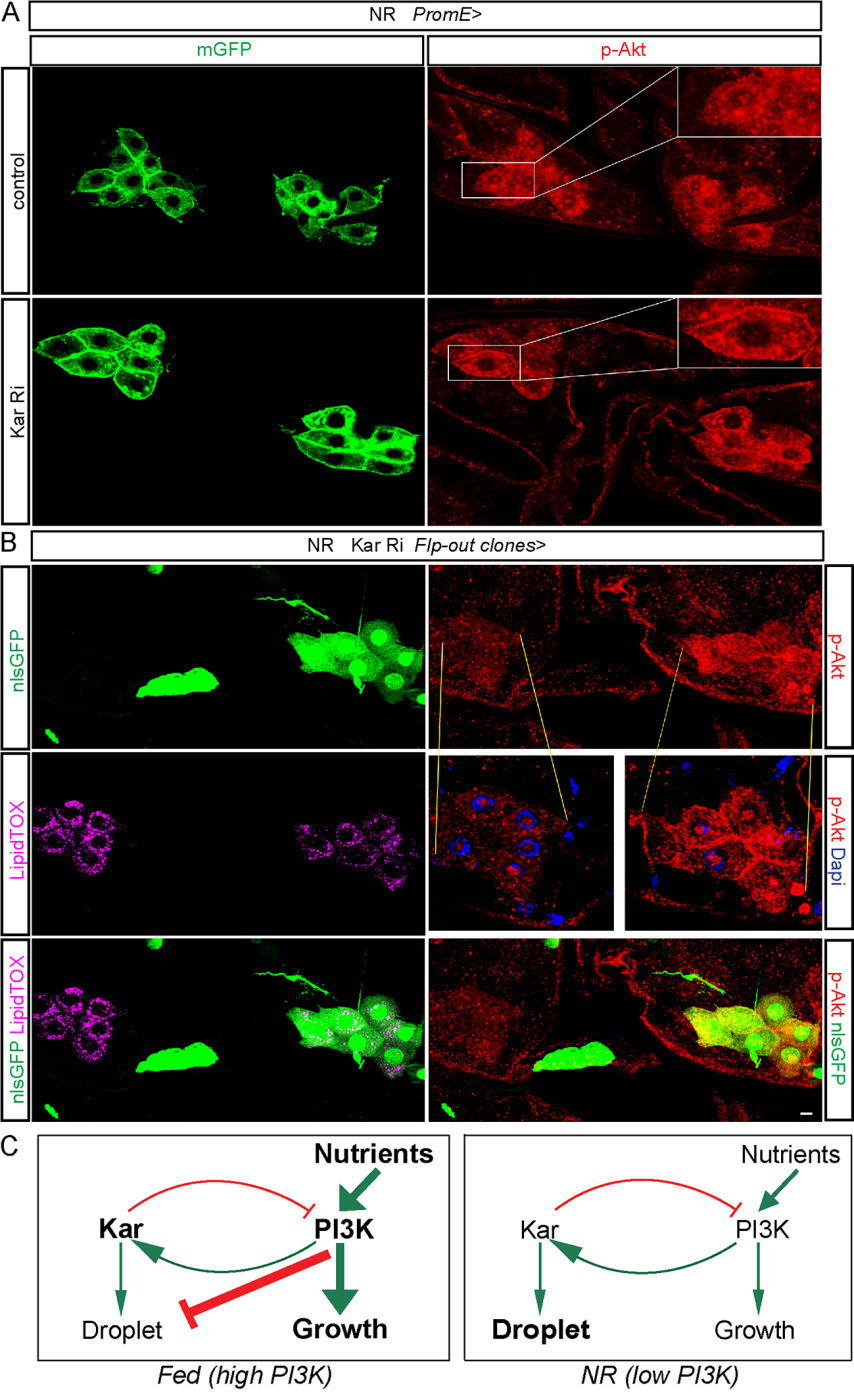
Kar is required to dampen the expression of membrane phospho-Akt. (A) Oenocyte clusters from NR larvae expressing *Kar* RNAi (*PromE>Kar RNAi*) and marked with GFP (mGFP) display higher intensity P-Akt staining than those from NR larvae of a control genotype. Single confocal sections are shown and insets correspond to a higher magnification of the boxed region. (B) The three rows of panels show two oenocyte clusters from a single NR larva, one of which is a Flp-out clone for Kar RNAi (marked with nlsGFP) that includes all oenocytes of one cluster. The nlsGFP-positive oenocyte cluster displays a decrease in lipid droplets (LipidTOX) and an increase in membrane p-Akt staining, compared to its neighboring control nlsGFP-negative oenocyte cluster. Nuclei are marked with DAPI and the scale bar is 10 μm. (C) Proposed model for the cross regulation between Kar and PI3K signaling in oenocytes. Diagrams depict the genetic interactions between Kar and PI3K signaling that balance cell growth and lipid droplet induction in fed (left panel) and nutrient restricted (right panel) states. In the fed state, cell growth predominates over lipid droplets as dietary nutrients stimulate high PI3K activity, which is prevented from becoming even higher by Kar mediated negative feedback, but also suppresses lipid droplet induction. In the nutrient restricted state, lipid droplets predominate over cell growth as Kar remains active, albeit at lower expression, but PI3K signaling is insufficient to promote substantial cell growth or to inhibit the lipid droplet induction process. Note that this model does not explain the genetic interactions underlying the aberrant accumulation of oenocyte lipid droplets in the fed state, as observed following Acc or Kar knockdown. Arrows indicate genetic interactions that are not necessarily mediated by the known enzymatic activities of the proteins (see text for details).

To investigate further the regulatory relationship between Kar, FarO and PI3K in larval oenocytes, we used immunostaining for phospho-AKT. No significant change in membrane phospho-Akt levels was detected following FarO knockdown in NR larvae, suggesting that it functions downstream or in parallel to PI3K during the negative regulation of oenocyte growth (S8A Fig). Importantly, Kar knockdown using either the *PromE-GAL4* or the Flp-out clonal method gave a moderate and reproducible increase in membrane phospho-Akt expression during NR (Figs 7A, 7B, S8B and S8C). Together with the genetic interaction analysis, these phospho-Akt results demonstrate that Kar plays a key role in inhibiting PI3K signaling and thus suppressing the inappropriate overgrowth of oenocytes.

## Discussion

### PI3K regulation of fat body-oenocyte lipid metabolism

During starvation, internal levels of nutrients decrease and growth is attenuated in many larval tissues including the fat body and oenocytes [19, 46]. Our study now suggests that the decrease in PI3K signaling during starvation plays a physiological role in permitting neutral lipids to be lost from the fat body and concomitantly gained in oenocytes. For the fat body, decreased PI3K signaling is a necessary but not a sufficient condition for neutral lipid loss, indicating that other regulators must also be important for the starvation response in this tissue. For larval oenocytes, reduced PI3K signaling is not only needed for lipid droplet induction during NR but it can also trigger inappropriate lipid droplets in the fed state. Hence, different tissue-specific responses to PI3K signaling may be an important component of the mechanism regulating lipid metabolism in the larval fat body-oenocyte axis.

It has been reported that starvation induces lipid droplets in adult oenocytes [42], as is known to be the case for larval oenocytes [35]. In contrast to our current findings in larvae, it was concluded in adult flies that starvation increases oenocyte PI3K signaling and that lipid droplet induction requires Insulin-like receptor signaling in oenocytes, triggered by Ilp6 secreted from the adult fat body [42]. For larval oenocytes, however, the lipid droplet induction mechanism is very different as our study shows that it is compromised by high PI3K signaling and does not require Ilp6.

### Oenocyte lipid droplet biogenesis requires VLCFA and hydrocarbon enzymes

This study reveals that genes metabolizing fatty acids (Acc), triglycerides (Dgat1), VLCFAs (FarO and possibly Kar) and hydrocarbons (Cyp4g1 and Cpr) are all required for maximal lipid droplet induction in larval oenocytes during NR. For Acc and Kar, this starvation function in lipid droplet induction adds to their previously known roles in waterproofing the larval tracheal system [36]. Our genetic analyses also suggest that derivatives of long and very long chain fatty acids, including hydrocarbons, could either regulate the induction of oenocyte lipid droplets or be part of their neutral lipid cargo. The fat composition of larval oenocyte lipid droplets is, however, not yet clear due to the inherent technical difficulty of obtaining enough purified material for meaningful lipidomic analysis. Given that the accumulation of as yet unidentified neutral lipids in larval oenocytes reflects increased fatty acid release from the fat body [35], it is tempting to speculate that they may play an important metabolic role during starvation. Although the physiological functions of oenocyte lipid droplets have not yet been clearly demonstrated in larvae, oenocyte lipid uptake in adult flies is thought to play an important role in promoting lipid turnover and survival during starvation [42].

### Kar mediates a PI3K negative feedback loop during oenocyte growth

An unexpected finding of this study is that two lipid dehydrogenase/reductase enzymes, Kar and FarO, regulate the balance between lipid droplets and the cell size of oenocytes. Knockdown of either enzyme blocks droplet induction and also increases cell size. Four other genes required for the induction of lipid droplets (Acc, Dgat1, Cyp4g1 and Cpr) did not detectably influence oenocyte size, arguing that blocking lipid droplet induction *per se* does not automatically lead to an increase in cell growth. Oenocyte size regulation therefore appears to be a selective property of a subset of lipid metabolic enzymes rather than all enzymes that synthesize/modify fatty acids. Kar is reported to be a 3-ketoacyl-CoA reductase for the elongation of long chain fatty acids into VLCFAs and it is known to be required in oenocytes to produce their derivatives, the cuticular hydrocarbons [34, 36]. A recent study, however, provides evidence that Kar, also known as Spidey, regulates ecdysteroid levels in larvae and so could function as a steroid dehydrogenase rather than a ketoacyl-CoA reductase [47]. For FarO, we demonstrated biochemically that it is a *bona fide* VLC fatty acyl-CoA reductase that reduces VLCFA-CoA esters into their corresponding VLCF alcohols. It remains to be established, however, whether or not the functions of Kar or FarO in cell growth require their reductase/dehydrogenase catalytic activities. Intriguingly, we found that Kar is required for the induction of oenocyte lipid droplets during starvation, despite a decrease in its protein levels. Genetic analysis indicates that the lower levels of Kar during NR are compatible with its function in lipid droplet induction. Presumably less Kar is also needed during NR for its other function in limiting PI3K activity, which is itself much lower during NR than in the fed state. The Kar decrease during NR can be mimicked in fed larvae by blocking oenocyte PI3K signaling, indicating that PI3K is a positive regulator of Kar expression. Our study also provides two lines of evidence for the reciprocal regulation i.e. Kar-dependent repression of PI3K signaling. Hence, the increase in oenocyte growth following Kar knockdown requires PI3K activity and membrane phospho-Akt levels are elevated by Kar knockdown. The existence of such cross-regulation supports a model in which a PI3K negative feedback loop mediated by Kar prevents excessive oenocyte growth (Fig 7C). So why does PI3K negative feedback mediated by Kar exist in some cell types (e.g oenocytes) but apparently not in others (e.g fat body cells)? One possibility is that this regulatory coupling is only important in those cell types that express high levels of Kar and dedicate a large fraction of their energy resources to metabolizing its substrates. Kar's role and indeed that of FarO could therefore be to ensure that critical physiological functions of oenocytes requiring VLCFA or ecdysteroid metabolism are not sacrificed at the expense of growth. Future biochemical and biophysical studies will be needed in order to elucidate the mechanisms by which Kar and FarO suppress oenocyte growth. Regulation of PI3K signaling at the level of membrane p-Akt was detected for Kar, although not for FarO. In principle, Kar could regulate PI3K signaling via effects upon ecdysone signaling and/or by changing the composition of the membrane where PI3K is active. With regard to the latter possibility, it is interesting that studies in various other biological contexts have shown that VLCFAs can modulate the activity of the PI3K signaling pathway. For example, docosahexenoic acid, a polyunsaturated VLCFA, inhibits PI3K signaling and the growth of prostate and colon cancer cells whereas it can stimulate PI3K signaling and the survival of neuronal cells [48–50]. Future studies will reveal whether oenocyte-like Kar regulation of PI3K exists in these contexts or in other tissues known to synthesize large quantities of VLCFAs and other specialized lipids such as those found in the mammalian liver and skin.

## Materials and Methods

### *Drosophila* strains and larval analysis

*Drosophila* were raised at 25°C using a standard cornmeal/agar diet unless otherwise stated. Larvae were staged at the L2/L3 molt according to morphology and early L3 larvae were either dissected immediately (the Fed_48_ group) or transferred for 18hr to PBS/1% low melting point agarose (the NR_66_ group) or for 18h to food (the Fed_66_ group) before dissection (Fig 1). For all RNAi experiments, early L1 larvae were transferred to 29°C until dissection. For tracheal flooding assays, larvae were transferred 6-8hr after the L2/L3 molt from food to PBS in a petri dish and maintained submerged for 15 min using a wire mesh. The numbers of larvae with liquid in the main tracheal trunks were then scored. The oenocyte specific driver line *PromE-Gal4*, *UAS-CD8*::*GFP* [31] is expressed from embryonic stage 17 onwards. Key results with *PromE-GAL4* were confirmed using a second oenocyte-specific GAL4 driver line *Cee-GAL4* (*C**yp4g1-Gal4*; *e**lav-Gal80*; *e**lav-Gal80*), which is strongly expressed from the L1 stage in oenocytes and salivary glands (and weakly in trachea) and was generated by combining a copy of *elav*-Gal80 on the 2nd and on the 3rd chromosomes (gifts of Matthias Llandgraf) with *Cyp4g1*-Gal4^*NP6073*^ (DGRC, Kyoto) to suppress unwanted neural Gal4 activity. To induce flp-out clones, *hs-flp*; *actin5C>CD2>Gal4* flies were crossed to the relevant *UAS* line and early L1 progeny were heat shocked at 37°C for 8 min. The larval progeny of GAL4 or UAS lines crossed to *w*^*1118*^ were used as controls. Oenocyte-less larvae were generated by crossing *PromE-Gal4* to a *UAS-reaper* line [35]. Inducible RNA-interference (*UAS-RNAi*) lines for the genes *CG11198*^*KK108631*^ (*Acc*), *CG11198*^*GD8105*^ (*Acc*), CG1444^GD40949^ (*Kar*), CG1444^1444R-2^ (*Kar*), CG18031^GD30220^ (*FarO)*, CG31991^*GD6367*^ (*DGAT1*, *midway*), CG11567^GD46715^ (*Cpr*), CG11567^GD44232^ (*Cpr*), CG11567^KK107422^ (*Cpr*) and CG14049^GD45218^ (*Ilp6*) were obtained from VDRC and NIG-FLY. For the generation of inducible intron-spliced snapback *UAS-Cyp4g1* RNAi lines (O-194 and O-196), the cDNA clone GH05567 was used as a template and a 330 bp *Cyp4g1* fragment amplified with Pfu DNA polymerase using the following pairs of 5’ tagged primers: GAGTACTAGTAAGAGGAGTCACGTGCGATTGTTG and GTTGACTAGTGCGAAGACTTTAGCCTGGATG. The PCR fragment of *Cyp4g1* was cloned as an inverted repeat into the *UAS-RNAi* vector pWIZ [51] and introduced in *yw* hosts by P-element-mediated transformation [52]. With the conditions used, *PromE-GAL4* produced pupal/adult lethality when crossed with *UAS-RNAi* lines for *Acc*, *Kar*, *Dgat1*, *Cyp4g1*, *Cpr* or *Dp110*^*DN*^ but not for *FarO*, *Dp110* or overexpression of *Lsd2*. *Cyp4g1*^*Δ4*^ is a protein-null allele [35] and hemizygous mutant males were compared to *Cyp4g1*^*Δ4*^*/FM7c* female controls. Other stocks used were *Df(1)Ilp6* [53], *Cg-GAL4;UAS-Dicer2*, *UAS-nlsGFP*, *UAS-Dp110DN*^*A2860C*^, *UAS-Dp110* [54], *UAS-myrAkt1* [55], *tGPH* [39], *UAS-Lsd2* [line RKF391, 56]. Stocks obtained from the Bloomington *Drosophila* Stock Center (NIH P40OD018537) were used in this study.

### Lipid droplet staining

Oil Red O staining was performed as previously described [35] and images were acquired using a Zeiss Axiophot 2 compound microscope. LipidTOX staining was performed after immunostaining: tissues were washed with PBS + 0.3% Triton. Then washed several times with PBS to remove the detergent and incubated O/N at 4°C with LipidTOX (HCS LipidTOX Red or Deep Red Neutral Lipid Stain, Life Technologies) at 1:1000 dilution.

### Immunostaining

Larval pelts, cut along the dorsal side, were cleaned of internal tissues and immobilized in PBS cuticle face down with insect pins on silicone elastomer (Sylgard, Dow Corning) polymerized in a small petri dish. All subsequent reaction steps were done on pinned out pelts in the Sylgard dish, using a horizontal shaker. Pelts were then fixed in 4% formaldehyde (or 10% formaldehyde for P-Akt stainings) for 20 min. After permeabilization in PBST (PBS/0.1% TritonX-100) tissues were blocked for 1.5 hr in PBST containing 0.1% BSA, 10% NGS, then primary antibodies were added in block solution and incubated overnight at 4°C. After extensive washes, block solution containing secondary antibodies was added for 2 hr at room temperature, and then washed extensively. Washes (5x20 min) were in PBS/0.1% TritonX-100/0.1% BSA after incubation with primary and secondary antibodies. For P-Akt antibodies, samples were postfixed for 20 min in 4% formaldehyde. Pelts were then washed in PBST and mounted with the cuticle facing up in Vectashield (Vector Laboratories). Primary antibodies were used at the following dilutions: Acc (1:50 cat#3662 Cell Signaling Technology), rabbit anti-Foxo (1:500, gift of P. Leopold), P-Akt Ser505 (1:50, cat#4054 Cell Signaling Technology), mouse anti-Cyp4g1 (1:1000, 433-43-2e, gift of S. Kennel). Secondary goat antibodies used were Alexa Fluor F(ab')2 fragments (1: 400, Invitrogen) and streptavidin-A555 (1ug/mL, Molecular Probes). Nuclear labeling was performed by incubating the tissues with Dapi (1:1000 Sigma). Images were collected on a Nikon Digital Eclipse C1 confocal microscope. Affinity purified rabbit anti-Kar antibodies (used at 1:1000) and rabbit anti-Cpr antibodies (used at 1:500) were generated by Cambridge Research Biochemicals via immunization with the peptides LGTRKRALRRLAKEQ (307–321, reference protein sequence AAF46291) and YLKNKQPQGSEEVKV (497–511, reference protein sequence AAF52367) respectively. RNAi knockdown efficiencies for Acc, Cpr, Cyp4g1 and Kar were quantified in ImageJ from maximum intensity z-stack projections by measuring mean pixel intensity across an oenocyte cluster, followed by subtraction of background as the mean pixel intensity measured in the area surrounding each cluster. Relative lipid content was quantified from maximum intensity z-stack projections of LipidTOX stainings of fat body cells and oenocytes. Mean pixel intensities across the entire fat body cell or oenocyte cluster were calculated using the 'integrated density' value of the analysis measurement tool in Photoshop. Cell areas were manually selected according to CD8::GFP expression using the magnetic lasso tool. Similarly, mean pixel intensities for membrane p-Akt staining were quantified from single confocal sections by manually selecting the CD8::GFP positive membrane region of oenocytes.

### EdU incorporation in vivo

Early L2 larvae were transferred to NR medium (PBS/0.5% low melting point agarose) for 12 hr and then removed, washed, and transferred to NR medium containing 0.1 mM EdU for 24 hr where larvae were seen “feeding” on the medium. Then larvae were dissected, fixed and EdU was detected using the Click-iT EdU Alexa Fluor 555 Imaging Kit (Life Technologies).

### Oenocyte cell and nuclear volumes

Oenocyte cell volumes were measured from 1–2 oenocyte clusters from 5 different larvae per genotype. Oenocyte clusters expressing a CD8::GFP reporter were scanned on a Nikon Digital Eclipse C1 confocal microscope. The Z-stack was adjusted to cover the entire cluster and sections spaced 2 μm apart were collected in three channels. The channels were separated using ImageJ software (http://imagej.nih.gov/ij/) and the GFP channel was imported to Amira software (Visage Imaging, Inc.) for 3D volume analysis. The clusters were automatically segmented followed by manual verification of the segmentation process and the volume of each segmented cluster was calculated. The settings of the entire procedure were kept identical for all scanned clusters. Nuclear volumes were measured as above on individually segmented nuclei from DAPI stained images of 1 oenocyte cluster from 12 different larvae of each genotype. For fat body Flp-out clones, area measurements were calculated using the analysis measurement tool of Photoshop (Adobe Systems) with manual segmentation of GFP expressing cells using the magnetic lasso tool. For oenocyte Flp-out clones, volume measurements used Amira to segment the whole cluster automatically but with manual verification, followed by manual division into GFP-positive and GFP-negative cells. Volumes of GFP-positive cells were normalized to the mean volume of all GFP-negative oenocytes from the same cluster.

### Production of recombinant FarO

The FarO ORF (clone LP02712, DGRC Bloomington) was subcloned into pENTR (Invitrogen), modified to remove the *Nco*I site [57]. The ORF was amplified by PCR using InFusF1 and InFusR1 linker primers to attach 15 nt overlapping the pENTR insertion site. pENTR was amplified using pENTR F4 and pENTR R5 primers. All PCR reactions used proof-reading polymerase (Pfu Ultra II HS; Agilent). Products were purified from agarose gels, joined by Gibson assembly using the Infusion HD cloning kit (Clontech, Mountainview, CA), and completely sequenced at the Nevada Genomics Center to verify integrity. The ORF was then transferred into Baculo-Direct baculoviral DNA using LR recombinase (Invitrogen), and transfected into Sf9 cells by standard methods [57]. A high titre P3 viral stock was produced by successive amplification of P1 and P2 stocks, and re-confirmed by sequencing. To produce recombinant FarO in Sf9 cells, liquid cultures were seeded at a concentration of 0.8x10^6^ cells/ml with 10% fetal bovine serum in SF900 unsupplemented media (Life technologies). Cultures were incubated at 27°C with shaking at 1,300 rpm on orbital shakers for 72 hr and centrifuged at 3000 rpm in the Beckman GS-6R centrifuge for 10 min at 4°C. The supernatant was discarded and the pellet was washed twice by successive resuspension in 5 ml of 100 mM Tris-HCl pH 7. The washed pellet was resuspended in cell lysis buffer [(CLB); 10 ml 100 mM Tris-HCl pH 7 with the addition of 100 μM DTT, 0.5 mM PMSF and 10 μl of protease inhibitor cocktail (Sigma)]. Pelleted cells were resuspended in 3ml CLB and sonicated using a Branson hand held sonifier (VWR Scientific) with 15 one second bursts repeated 3 times. 1ml aliquots were separated and microcentrifuged at 13,000 rpm for 20 min at 4°C. Supernatants were either used directly for functional assays or for microsomal preparations. Microsomes were prepared by centrifuging the lysate supernatant at 53,000 rpm in a Beckman Optima MaxE ultracentrifuge for 1 h at 4°C, removing the supernatant, and resuspending the microsomal pellet in cell lysis buffer. Genscript (Piscataway, NJ) was contracted to produce rabbit antisera to a FarO peptide (amino acids 412–425). FarO production in Sf9 cells was measured by Western blotting of microsomal and supernatant fractions. Recombinant housefly cytochrome P450 reductase (CPR) was similarly produced as described previously [57].

### FarO functional assays

Recombinant FarO and CPR were assayed for reductase activity by incubating infected Sf9 cell lysate preparations with NAD(P)H and fatty acid (Sigma-Aldrich) or fatty acyl-CoA (Avanti Polar Lipids) substrates. Briefly, cell lysate supernatants in CLB were incubated in 100mM Tris HCl pH 7.0, 100 μM DTT, 0.5 mM PMSF and 10 μl of protease inhibitor cocktail (Sigma) supplemented with 120–150 μM 26:0–CoA or 24:0–CoA and 2.3 mM NADH (Fisher Scientific) or NADPH (Sigma Aldrich). All listed concentrations are final in 600–1000 μl reaction volumes. Samples were incubated at 30°C for 2 h and then extracted twice with hexane:ether (50/50, v/v) into glass vials. Samples were dried down to completion under N_2_ gas, resuspended in pure hexane and analyzed by gas chromatography using either a DB-5 column (Agilent) or a Shimadzu non-polar polysiloxane column, (catalog number: 220-94536-01, phase: SHR5XLB) with the following profile: injector temperature 150°C, FID temperature 300°C; program:160°C for 0.2 min, ramp to 265°C at 15°C/min, ramp to 295°C at 5°C/min, and hold for 5 min at 295°C.

## Supporting Information

S1 Fig Quantitations of neutral lipid content. The relative lipid contents, calculated from neutral LipidTOX stainings, for experiments shown in the main Figures. (A,B) Flp-out clones in the fat body expressing Dp110 in NR (A) or Dp110^DN^ in Fed_48_ larvae (B). (C-E) *PromE-GAL4* controls in Fed_48_ and NR larvae (C) and *PromE-GAL4* driven expression of Dp110 or Dp110^DN^ in Fed_48_ (D) and NR (E) larvae. (F,G) *PromE-GAL4* driven expression of RNAi for Acc, Dgat1, Cyp4g1 and Cpr, or of Lsd2 overexpression in Fed_48_ (F) and NR (G) larvae. (H) *Cyp4g1*^*Δ4*^ mutant NR larvae. (I,J) *PromE-GAL4* driven expression of RNAi for Kar or FarO in Fed_48_ (I) and NR (J) larvae. Quantifications in (A-E) correspond to Fig 1, (F-H) correspond to Fig 2 and (I,J) correspond to Fig 4. In this and subsequent graphs, error bars represent 1 s.d. and asterisks show statistical significance in Student t tests (*p<0.05, and **p<0.001), compared to the one fold control condition unless otherwise indicated
(TIF)

S2 Fig Quantitations of cell size and membrane p-Akt expression. (A, B) Fat body cell areas in Flp-out clones. Dp110 overexpression increases cell size relative to controls in NR larvae (A) and Dp110^DN^ expression decreases cell size relative to controls in Fed_48_ larvae (B). (C, D) Quantitation of Oenocyte membrane p-Akt expression. Membrane p-Akt expression increases significantly in Fed_48_ (C) or NR (D) larvae following *PromE-GAL4* driven Dp110 overexpression whereas the decreases following Dp110^DN^ expression are not statistically significant. (E-G) Relative oenocyte volumes in Fed_48_ (E, G) or NR (F) larvae significantly increase following *PromE-GAL4* driven Dp110 overexpression or Flp-out clonal expression of myrAkt. Oenocyte volumes significantly decrease following *PromE-GAL4* or Flp-out clonal expression of Dp110^DN^.
(TIF)

S3 Fig NR represses PI3K signaling more strongly in fat body than oenocytes. Panels in top two rows show endogenous FoxO expression in fat body (left) and oenocytes (right, marked with *PromE>mGFP*) from Fed_48_ and NR_66_ larvae. Redistribution of FoxO from the cytoplasm to the nucleus (associated with decreased PI3K signaling) during NR is more pronounced in the fat body than in oenocytes. The nuclei of NR oenocytes (white arrowhead) express less FoxO than the nuclei of neighboring NR epidermal cells (yellow arrowhead). Panels in the bottom row show expression of the *tGPH* reporter for PI3K activity in fat body (left) and oenocytes (right) from Fed_48_ and NR_66_ larvae. Membrane expression of EGFP fused to the pleckstrin homology domain of Grp1 (a readout for PI3K activity in some but not in all cell types) is clearly decreased during NR in fat body but in oenocytes the NR change is less noticeable. Scale bar is 10 μm.
(TIF)

S4 Fig Ilp6 is not required for NR induction of lipid droplets in larval oenocytes. Clusters of oenocytes from NR larvae, marked with streptavidin-A555 and showing lipid droplets (LipidTOX) and nuclei (DAPI). (A) Larvae deficient for Ilp6 (*Df(1)Ilp6*) show lipid droplet induction similar to controls (*w*^*1118*^). (B) Larvae with fat body-specific RNAi knockdown of Ilp6 (*Cg>Ilp6 Ri*) show lipid droplet induction similar to controls (*Cg-GAL4*). Scale bar is 20 μm. (C) Most *Acc*, *Kar*, *Cpr* and *Cyp4g1* RNAi larvae retain watertight trachea. Graph shows the percentage of larvae of various genotypes showing tracheal flooding at the early L3 stage. Asterisks indicates p<0.01. Each larval genotype carried the oenocyte-specific driver Pro-*mE-GAL4* and the respective *UAS-RNAi* transgene indicated. The control genotype used was *PromE-GAL4 crossed to w*^*1118*^ and "oenocyte-less" refers to *PromE>reaper*.
(TIF)

S5 Fig Quantitation of protein knockdowns with Acc, Kar, Cyp4g1 and Cpr RNAi. (A) Antibody staining for Acc, Kar, Cyp4g1 and Cpr in oenocytes following *PromE-GAL4* driven RNAi in NR larvae. Scale bar is 20μm. (B) Staining for Kar in nlsGFP positive Flp-out clones expressing Kar Ri in oenocytes. Scale bar is 10μm. (C) Graph of relative immunostaining intensity for Kar protein in Kar Ri oenocyte Flp-out clones during NR. (D-G) Graphs of relative staining intensity in oenocytes for Acc (D), Kar (E), Cyp4g1 (F) and Cpr (G) proteins following *PromE-GAL4* driven RNAi knockdown of the corresponding genes in NR larvae.
(TIF)

S6 Fig FarO and Kar do not limit the size of oenocytes at the late larval stage. (A) Neither Kar nor FarO RNAi detectably alter lipid droplets in the fat body. Flp-out clones for Kar RNAi (top row) or FarO RNAi (bottom row), marked with nlsGFP, show no detectable change in lipid droplets (LipidTOX), nuclear size or cell size in NR larvae. Nuclei are marked with DAPI and the scale bar is 10μm. (B, C) Oenocyte FarO Ri Flp-out clones in NR larvae, marked by nlsGFP, show decreased LipidTOX staining (B) and significantly decreased relative neutral lipid content (C) than control neighboring cells. Oenocyte Kar expression is not altered in FarO Ri Flp-out clones. (D, E) FarO and Kar do not regulate oenocyte volumes at the late larval stage. *PromE-GAL4* mediated expression of *Kar* or FarO RNAi alone or in combination with *Dp110*^*DN*^ in Fed_66_ larvae. In contrast to Fed_48_ and NR_66_ larvae, oenocyte volumes in Fed_66_ larvae are not significantly altered by *Kar* or *FarO* RNAi, although they remain PI3K dependent.
(TIF)

S7 Fig Genetic interactions between FarO and PI3K. (A) In Fed_48_ larvae, *PromE>Dp110*^*DN*^ induces a low level of oenocyte lipid droplets that are suppressed in *PromE>FarO Ri Dp110*^*DN*^. In NR larvae, *PromE>Dp110*^*DN*^ does not noticeably alter lipid droplet induction and thus inhibition of NR droplet induction is similar in *PromE>FarO Ri* and in *PromE>FarO Ri Dp110*^*DN*^. In both Fed_48_ and NR larvae, oenocyte volume in *PromE>FarO Ri Dp110*^*DN*^ is intermediate between that of *PromE>FarO Ri* alone and *PromE>Dp110*^*DN*^ alone. Panels show single confocal sections and the scale bar is 10 μm. (B) Relative neutral lipid content of oenocytes from Fed_48_ larvae for the genotypes in panel A. Note that FarO is required for the increase in lipid droplets in fed larvae following PI3K inhibition. (C) Oenocyte volumes for the genotypes in panel A in Fed_48_ and NR_66_ larvae.
(TIF)

S8 Fig Kar but not FarO RNAi significantly increases oenocyte membrane p-Akt. (A, B) Quantitation of membrane p-Akt intensity, relative to the control genotype, in NR oenocytes expressing FarO RNAi (A) or Kar RNAi (B). Membrane p-Akt intensity increases 1.7 fold with Kar RNAi but does not change significantly with FarO RNAi. (C) Panels show p-Akt staining in an oenocyte cluster (marked with mGFP) from control and Kar knockdown (*PromE>Kar* RNAi) NR_66_ larvae. Kar RNAi knockdown is associated with increased membrane p-Akt expression. Scale bar is 10 μm.
(TIF)
