# Supplementary figures and images for: Drosophila Spidey/Kar Regulates Oenocyte Growth via PI3-Kinase Signaling
Source: PLoS Genet. 2016 Aug 8;12(8):e1006154. doi: 10.1371/journal.pgen.1006154 (PMC4976899; doi:10.1371/journal.pgen.1006154)

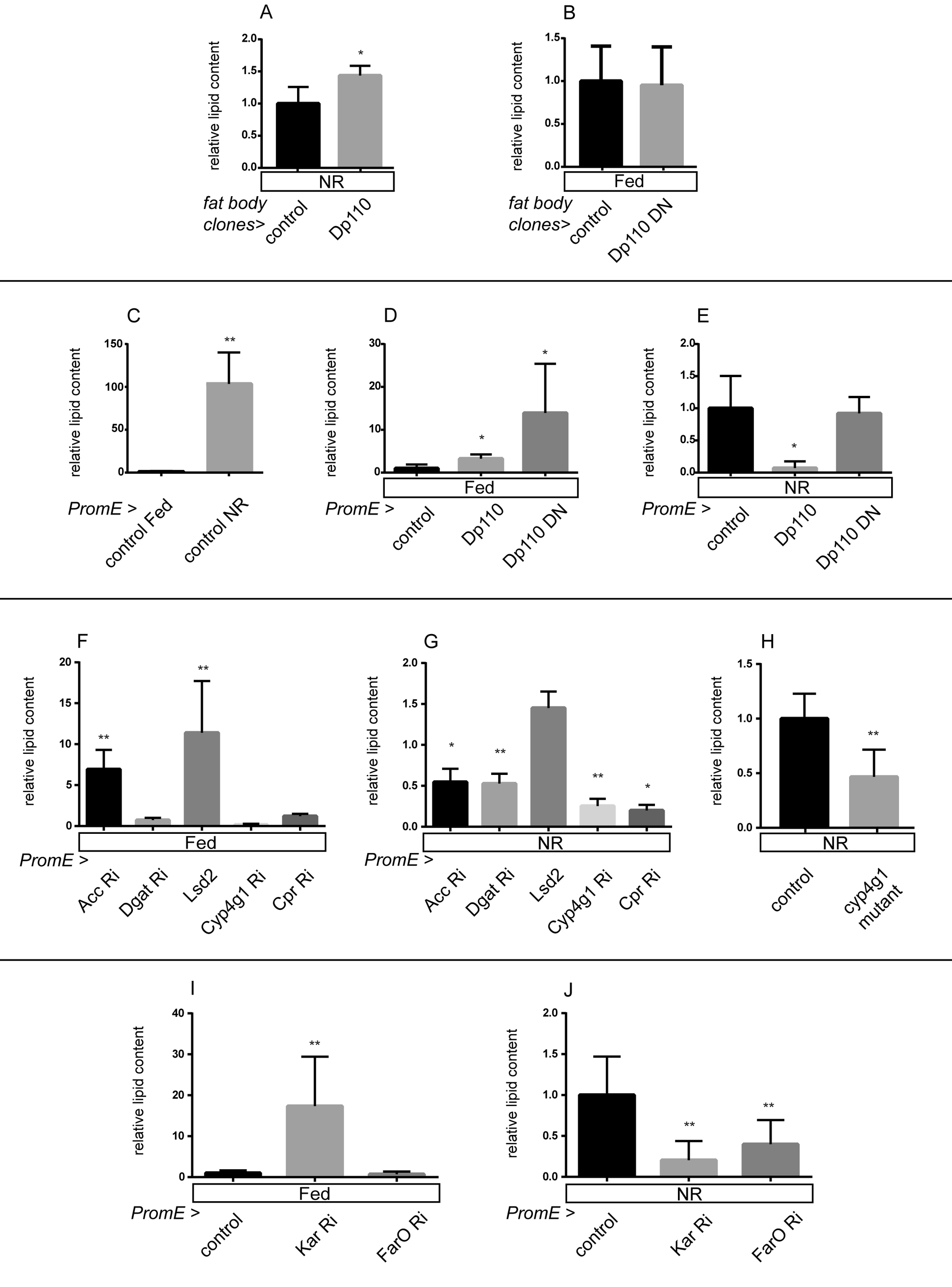

Supplement: S1 Fig — The relative lipid contents, calculated from neutral LipidTOX stainings, for experiments shown in the main Figures. (A,B) Flp-out clones in the fat body expressing Dp110 in NR (A) or Dp110DN in Fed48 larvae (B). (C-E) PromE-GAL4 controls in Fed48 and NR larvae (C) and PromE-GAL4 driven expression of Dp110 or Dp110DN in Fed48 (D) and NR (E) larvae. (F,G) PromE-GAL4 driven expression of RNAi for Acc, Dgat1, Cyp4g1 and Cpr, or of Lsd2 overexpression in Fed48 (F) and NR (G) larvae. (H) Cyp4g1Δ4 mutant NR larvae. (I,J) PromE-GAL4 driven expression of RNAi for Kar or FarO in Fed48 (I) and NR (J) larvae. Quantifications in (A-E) correspond to Fig 1, (F-H) correspond to Fig 2 and (I,J) correspond to Fig 4. In this and subsequent graphs, error bars represent 1 s.d. and asterisks show statistical significance in Student t tests (*p<0.05, and **p<0.001), compared to the one fold control condition unless otherwise indicated (TIF) [file pgen.1006154.s001.tif]

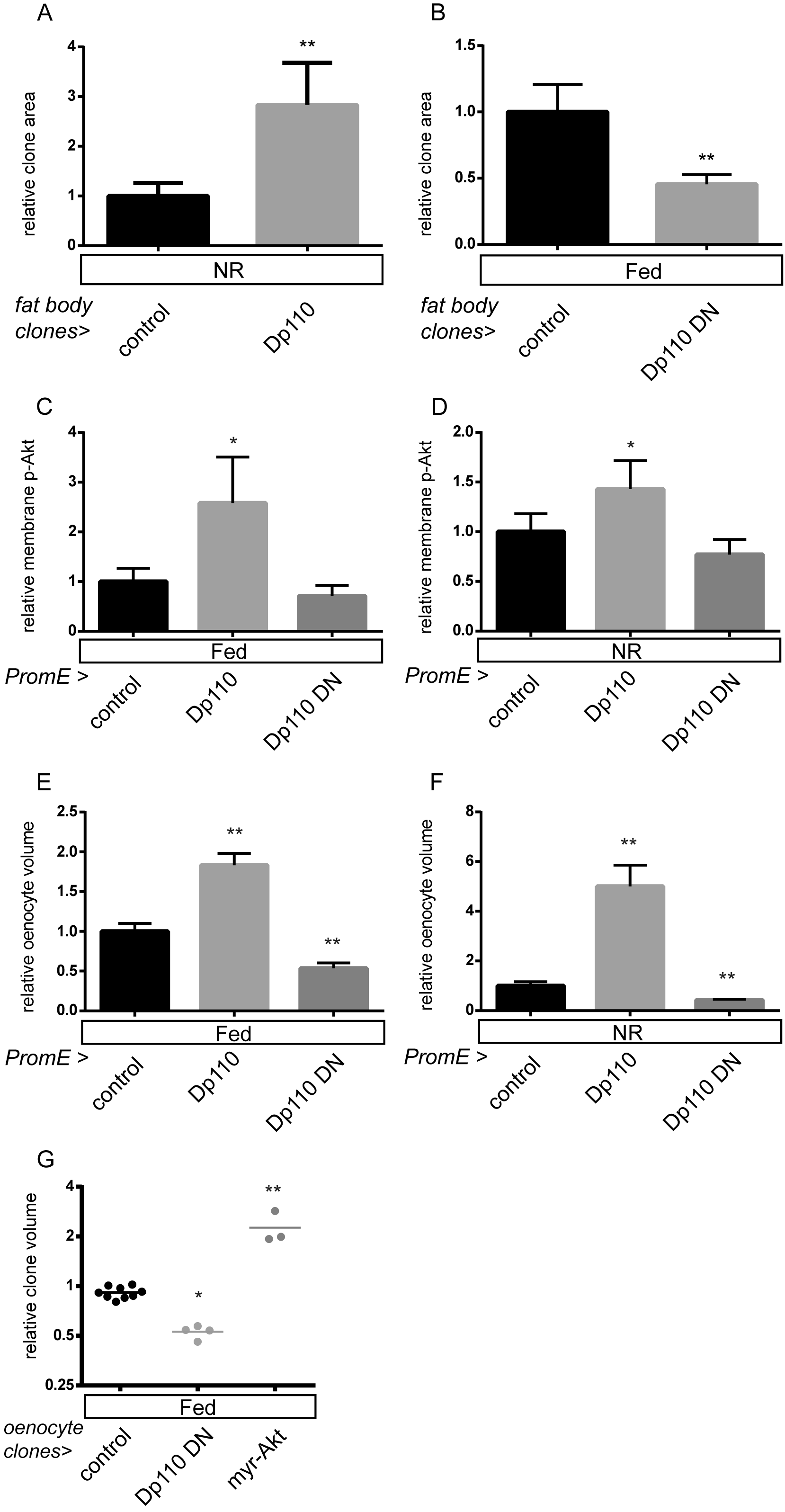

Supplement: S2 Fig — (A, B) Fat body cell areas in Flp-out clones. Dp110 overexpression increases cell size relative to controls in NR larvae (A) and Dp110DN expression decreases cell size relative to controls in Fed48 larvae (B). (C, D) Quantitation of Oenocyte membrane p-Akt expression. Membrane p-Akt expression increases significantly in Fed48 (C) or NR (D) larvae following PromE-GAL4 driven Dp110 overexpression whereas the decreases following Dp110DN expression are not statistically significant. (E-G) Relative oenocyte volumes in Fed48 (E, G) or NR (F) larvae significantly increase following PromE-GAL4 driven Dp110 overexpression or Flp-out clonal expression of myrAkt. Oenocyte volumes significantly decrease following PromE-GAL4 or Flp-out clonal expression of Dp110DN. (TIF) [file pgen.1006154.s002.tif]

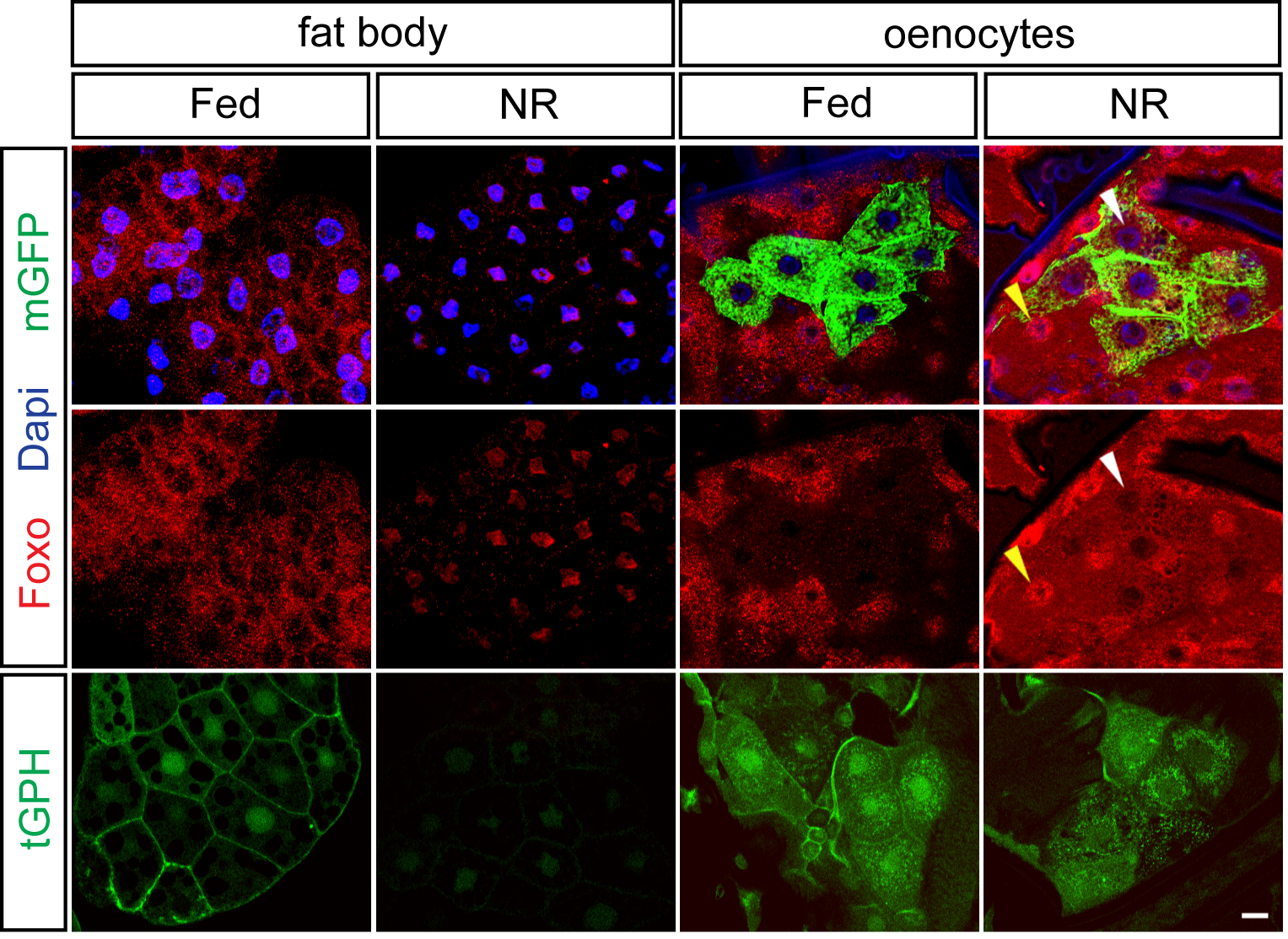

Supplement: S3 Fig — Panels in top two rows show endogenous FoxO expression in fat body (left) and oenocytes (right, marked with PromE>mGFP) from Fed48 and NR66 larvae. Redistribution of FoxO from the cytoplasm to the nucleus (associated with decreased PI3K signaling) during NR is more pronounced in the fat body than in oenocytes. The nuclei of NR oenocytes (white arrowhead) express less FoxO than the nuclei of neighboring NR epidermal cells (yellow arrowhead). Panels in the bottom row show expression of the tGPH reporter for PI3K activity in fat body (left) and oenocytes (right) from Fed48 and NR66 larvae. Membrane expression of EGFP fused to the pleckstrin homology domain of Grp1 (a readout for PI3K activity in some but not in all cell types) is clearly decreased during NR in fat body but in oenocytes the NR change is less noticeable. Scale bar is 10 μm. (TIF) [file pgen.1006154.s003.tif]

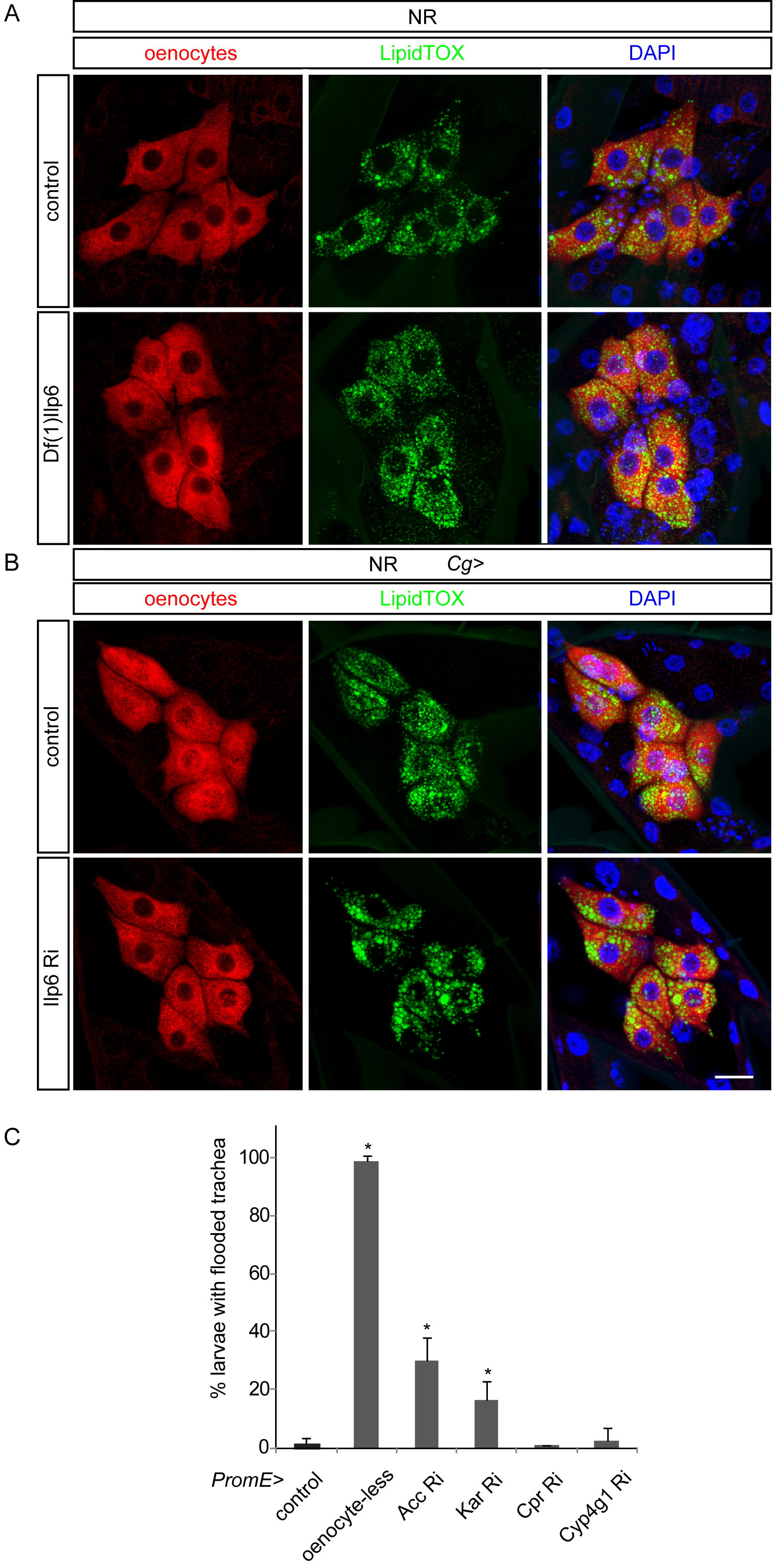

Supplement: S4 Fig — Clusters of oenocytes from NR larvae, marked with streptavidin-A555 and showing lipid droplets (LipidTOX) and nuclei (DAPI). (A) Larvae deficient for Ilp6 (Df(1)Ilp6) show lipid droplet induction similar to controls (w1118). (B) Larvae with fat body-specific RNAi knockdown of Ilp6 (Cg>Ilp6 Ri) show lipid droplet induction similar to controls (Cg-GAL4). Scale bar is 20 μm. (C) Most Acc, Kar, Cpr and Cyp4g1 RNAi larvae retain watertight trachea. Graph shows the percentage of larvae of various genotypes showing tracheal flooding at the early L3 stage. Asterisks indicates p<0.01. Each larval genotype carried the oenocyte-specific driver Pro-mE-GAL4 and the respective UAS-RNAi transgene indicated. The control genotype used was PromE-GAL4 crossed to w1118 and "oenocyte-less" refers to PromE>reaper. (TIF) [file pgen.1006154.s004.tif]

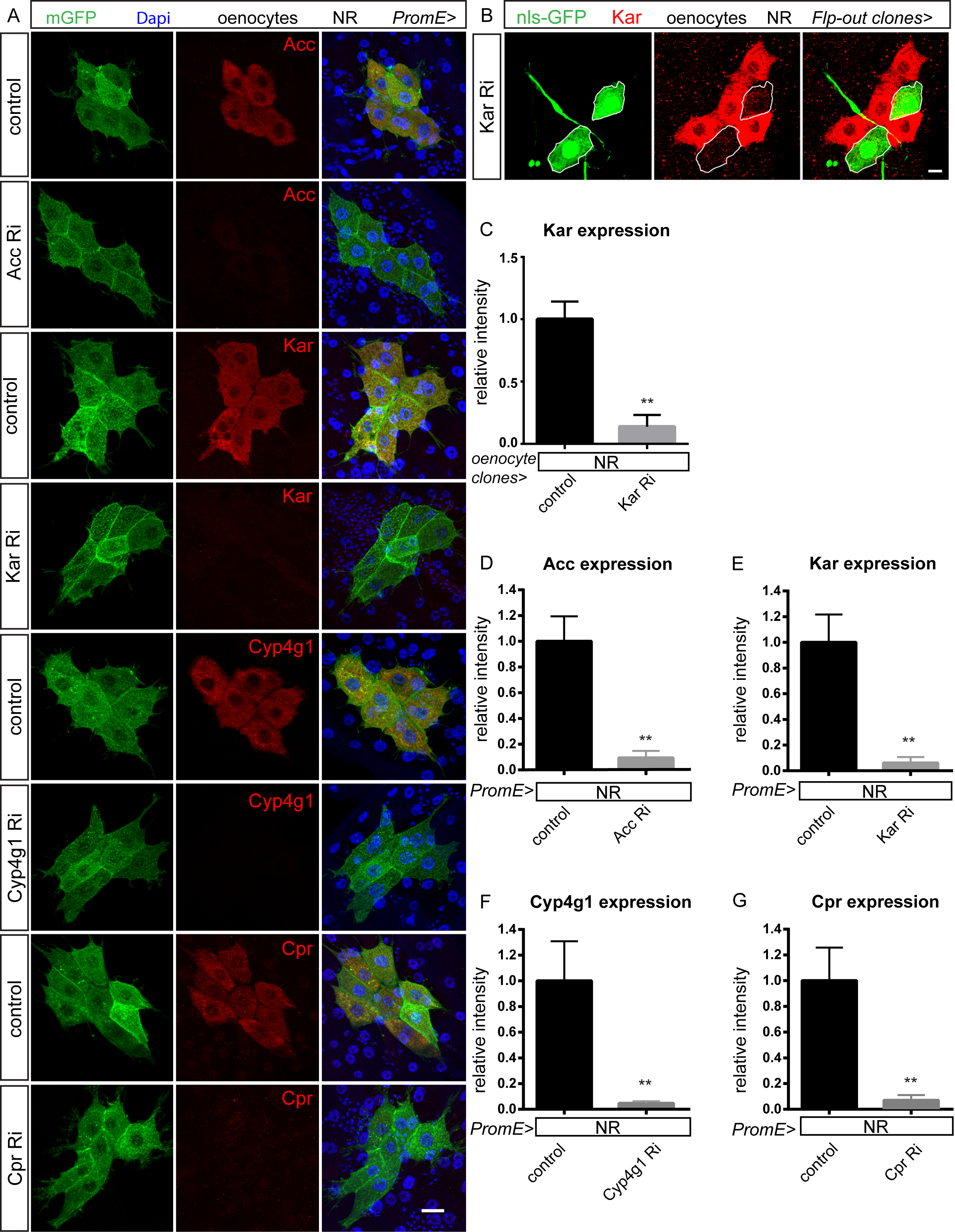

Supplement: S5 Fig — (A) Antibody staining for Acc, Kar, Cyp4g1 and Cpr in oenocytes following PromE-GAL4 driven RNAi in NR larvae. Scale bar is 20μm. (B) Staining for Kar in nlsGFP positive Flp-out clones expressing Kar Ri in oenocytes. Scale bar is 10μm. (C) Graph of relative immunostaining intensity for Kar protein in Kar Ri oenocyte Flp-out clones during NR. (D-G) Graphs of relative staining intensity in oenocytes for Acc (D), Kar (E), Cyp4g1 (F) and Cpr (G) proteins following PromE-GAL4 driven RNAi knockdown of the corresponding genes in NR larvae. (TIF) [file pgen.1006154.s005.tif]

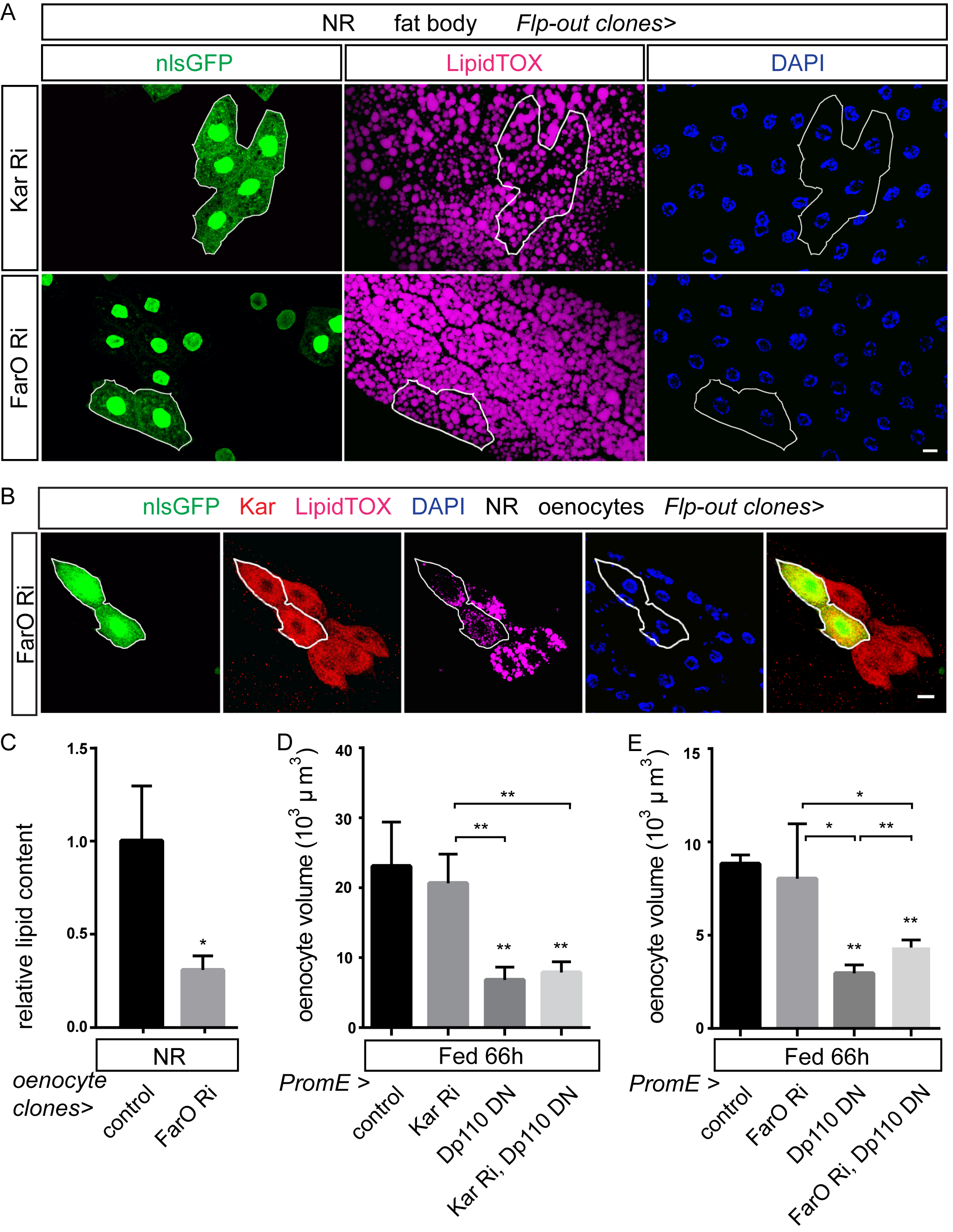

Supplement: S6 Fig — (A) Neither Kar nor FarO RNAi detectably alter lipid droplets in the fat body. Flp-out clones for Kar RNAi (top row) or FarO RNAi (bottom row), marked with nlsGFP, show no detectable change in lipid droplets (LipidTOX), nuclear size or cell size in NR larvae. Nuclei are marked with DAPI and the scale bar is 10μm. (B, C) Oenocyte FarO Ri Flp-out clones in NR larvae, marked by nlsGFP, show decreased LipidTOX staining (B) and significantly decreased relative neutral lipid content (C) than control neighboring cells. Oenocyte Kar expression is not altered in FarO Ri Flp-out clones. (D, E) FarO and Kar do not regulate oenocyte volumes at the late larval stage. PromE-GAL4 mediated expression of Kar or FarO RNAi alone or in combination with Dp110DN in Fed66 larvae. In contrast to Fed48 and NR66 larvae, oenocyte volumes in Fed66 larvae are not significantly altered by Kar or FarO RNAi, although they remain PI3K dependent. (TIF) [file pgen.1006154.s006.tif]

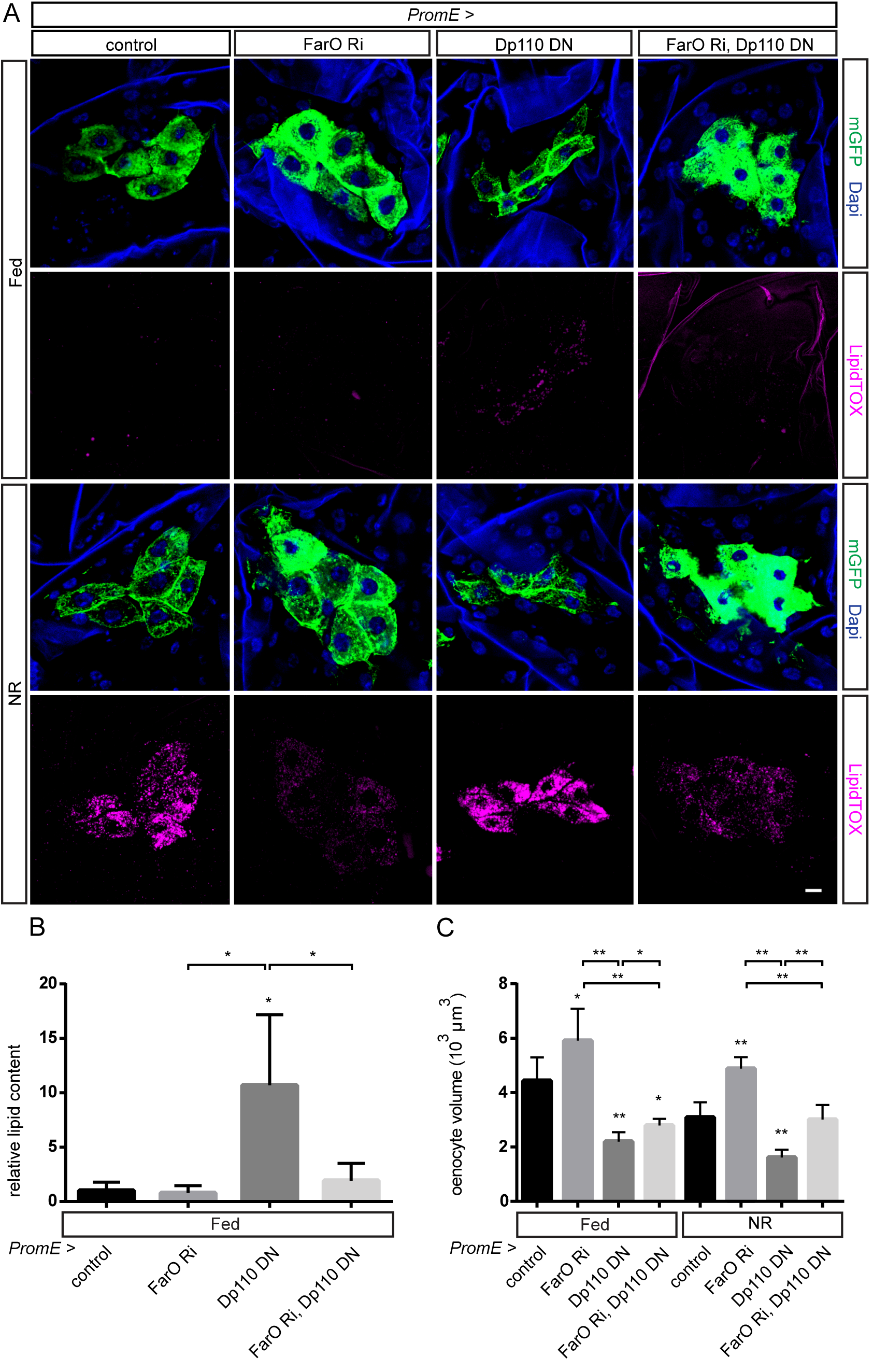

Supplement: S7 Fig — (A) In Fed48 larvae, PromE>Dp110DN induces a low level of oenocyte lipid droplets that are suppressed in PromE>FarO Ri Dp110DN. In NR larvae, PromE>Dp110DN does not noticeably alter lipid droplet induction and thus inhibition of NR droplet induction is similar in PromE>FarO Ri and in PromE>FarO Ri Dp110DN. In both Fed48 and NR larvae, oenocyte volume in PromE>FarO Ri Dp110DN is intermediate between that of PromE>FarO Ri alone and PromE>Dp110DN alone. Panels show single confocal sections and the scale bar is 10 μm. (B) Relative neutral lipid content of oenocytes from Fed48 larvae for the genotypes in panel A. Note that FarO is required for the increase in lipid droplets in fed larvae following PI3K inhibition. (C) Oenocyte volumes for the genotypes in panel A in Fed48 and NR66 larvae. (TIF) [file pgen.1006154.s007.tif]

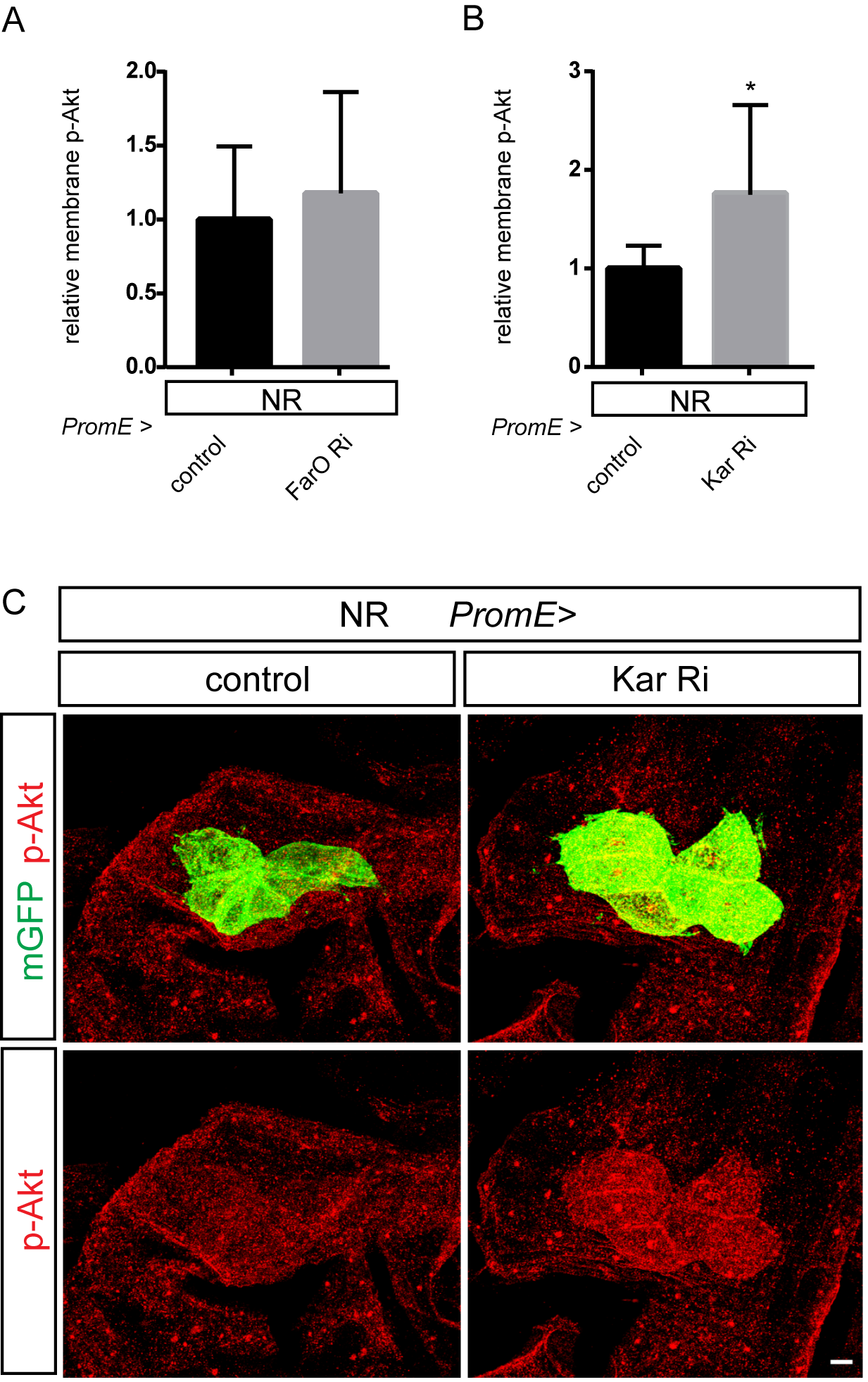

Supplement: S8 Fig — (A, B) Quantitation of membrane p-Akt intensity, relative to the control genotype, in NR oenocytes expressing FarO RNAi (A) or Kar RNAi (B). Membrane p-Akt intensity increases 1.7 fold with Kar RNAi but does not change significantly with FarO RNAi. (C) Panels show p-Akt staining in an oenocyte cluster (marked with mGFP) from control and Kar knockdown (PromE>Kar RNAi) NR66 larvae. Kar RNAi knockdown is associated with increased membrane p-Akt expression. Scale bar is 10 μm. (TIF) [file pgen.1006154.s008.tif]
